# Glucocorticoids Bind to SARS-CoV-2 S1 at Multiple Sites Causing Cooperative Inhibition of SARS-CoV-2 S1 Interaction With ACE2

**DOI:** 10.3389/fimmu.2022.906687

**Published:** 2022-06-15

**Authors:** Hassan Sarker, Rashmi Panigrahi, Eugenio Hardy, J. N. Mark Glover, Shokrollah Elahi, Carlos Fernandez-Patron

**Affiliations:** ^1^ Department of Biochemistry, Faculty of Medicine and Dentistry, University of Alberta, Edmonton, AB, Canada; ^2^ Center of Molecular Immunology, Havana, Cuba; ^3^ Department of Dentistry, Faculty of Medicine and Dentistry, University of Alberta, Edmonton, AB, Canada; ^4^ Department of Medical Microbiology and Immunology, University of Alberta, Edmonton, AB, Canada; ^5^ Department of Oncology, Faculty of Medicine and Dentistry, University of Alberta, Edmonton, AB, Canada

**Keywords:** glucocorticoids, SARS-CoV-2, ACE2 (angiotensin converting enzyme 2), innate immunity, COVID, coronavirus

## Abstract

Dexamethasone may reduce mortality in COVID-19 patients. Whether dexamethasone or endogenous glucocorticoids, such as cortisol, biochemically interact with SARS-CoV-2 spike 1 protein (S1), or its cellular receptor ACE2, is unknown. Using molecular dynamics (MD) simulations and binding energy calculations, we identified 162 druggable pockets in various conformational states of S1 and all possible binding pockets for cortisol and dexamethasone. Through biochemical binding studies, we confirmed that cortisol and dexamethasone bind to S1. Limited proteolysis and mass spectrometry analyses validated several MD identified binding pockets for cortisol and dexamethasone on S1. Interaction assays indicated that cortisol and dexamethasone separately and cooperatively disrupt S1 interaction with ACE2, through direct binding to S1, without affecting ACE2 catalytic activity. Cortisol disrupted the binding of the mutant S1 Beta variant (E484K, K417N, N501Y) to ACE2. Delta and Omicron variants are mutated in or near identified cortisol-binding pockets in S1, which may affect cortisol binding to them. In the presence of cortisol, we find increased inhibition of S1 binding to ACE2 by an anti-SARS-CoV-2 S1 human chimeric monoclonal antibody against the receptor binding domain. Whether glucocorticoid/S1 direct interaction is an innate defence mechanism that may have contributed to mild or asymptomatic SARS-CoV-2 infection deserves further investigation.

## Introduction

Coronaviruses (CoV) are enveloped viruses with a positive sense, single-stranded RNA genome that belong to the subfamily *Coronavirinae* of *Coronaviridae* viral family (1). Seven CoVs are known to infect humans and four of them are endemic human CoVs that cause common colds annually. At least three zoonotic CoVs have caused major outbreaks in humans: severe acute respiratory syndrome coronavirus-1 (SARS-CoV-1, which had an outbreak in 2002-2003), Middle East respiratory syndrome-coronavirus (MERS-CoV, which had an outbreak in 2012) and severe acute respiratory syndrome coronavirus-2 (SARS-CoV-2), whose outbreak is responsible for coronavirus disease 19 (COVID-19) - a pandemic with disease severity ranging from asymptomatic infection to severe pneumonia, acute respiratory distress and death (1, 2).

SARS-CoV-1, MERS-CoV and SARS-CoV-2 can infect humans through binding to target cell surface receptors such as angiotensin-converting enzyme 2 (ACE2). Binding to ACE2 is mediated *via* spike –a viral surface-expressed glycoprotein, which contains a receptor binding domain (RBD) through which these coronaviruses interact with ACE2 (2).

SARS-CoV-2 spike forms a trimeric protein located on the viral membrane and comprises a central helical stalk (S2 component) capped by a N-terminal S1 component (**Supplementary Figure 1**). Each S1 monomer of the spike trimer contains a large N-terminal domain (NTD), in addition to the RBD. Trimeric spike on the viral membrane exists in a ‘closed’ form, in which the RBDs cap the top of the S2 core and are inaccessible to ACE2 (3–5). However, spike can also adopt an ‘open’ form, in which one S1 component has opened exposing the RBD for ACE2 binding –this mechanism is captured in the cryogenic-electron microscopy (cryo-EM) structure (PDB: 6VSB, 6VYB) (3–9). It is thought that, for ACE2 engagement, the RBD undergoes structural movements between a receptor-inaccessible conformation and a receptor-accessible conformation. Further, cell entry requires spike priming by cellular proteases such as co-receptor transmembrane serine protease 2 (TMPRSS2) (2), which cleave spike at the S1/S2 site to facilitate fusion of viral and cellular membranes (4, 10). As the structural conformation of RBD is crucial for ACE2 engagement, molecules that perturb the structure of RBD have the potential to decrease RBD affinity for ACE2. Indeed, effective blockade of the SARS-CoV-2 spike interaction with ACE2 can be elicited by antibodies against the RBD such as those induced by viral infection or effective vaccines and found in the plasma of convalescent or vaccinated individuals (11, 12).

We hypothesized that non-antibody classes of biomolecules that bind spike at one or many sites can perturb the conformation of the RBD and, consequently, reduce the RBD affinity for ACE2. We tested our hypothesis for glucocorticoids owing to the huge physiological and clinical significance of this class of biomolecules and their synthetic analogues. In humans, the adrenal cortex produces more than 50 different glucocorticoid hormones which are subdivided into glucocorticoids (such as cortisol) and mineralocorticoids (such as aldosterone) (13). A number of synthetic glucocorticoids such as dexamethasone, prednisone and prednisolone have been applied for their anti-inflammatory or immune-suppressive actions (13, 14) in syndromes closely related to COVID-19, including SARS, MERS, severe influenza, and community-acquired pneumonia with various efficacies (15, 16). The United Kingdom-based Randomized Evaluation of COVID-19 Therapy trial found that dexamethasone 6 mg/d for 10 days results in reduction in 28-day mortality in patients receiving invasive mechanical ventilation at the time of randomization (17). Similar beneficial effects have been reported for various formulations of dexamethasone, hydrocortisone (cortisol), and methylprednisolone in reduction of mortality in COVID-19 patients requiring supplemental oxygen (15, 17, 18). However, the mechanisms underlying the beneficial effects of glucocorticoids in reduction of mortality in COVID-19 patients are unclear. Lack of knowledge on the specific mechanisms of action of glucocorticoids in the settings of SARS-CoV-2 infection prevents improving their efficacy and expanding their application to a broad spectrum of SARS-CoV-2 infected patients.

A possible interaction between dexamethasone and the RBD (6, 19) has been suggested (7). However, these studies are far from being conclusive and it remains to be established: (i) whether endogenous glucocorticoids as well as their synthetic analogues (like dexamethasone) can bind to the RBD at one or multiple sites, (ii) what the specific binding sites of glucocorticoids on the RBD are, (c) if glucocorticoids binding to the RBD reduces SARS-CoV-2 S1 affinity for ACE2. Mechanistic studies addressing this paucity of knowledge are warranted to better understand the SARS-CoV-2/host interactions and delineate new therapeutic interventions for reducing infectivity of SARS-CoV-2 and other coronaviruses that use spike to infect their host.

Here, we report that cortisol and dexamethasone (endogenous and synthetic glucocorticoids, respectively) can separately and cooperatively interact with SARS-CoV-2 S1 protein and effectively decrease the affinity of S1 for ACE2. Through molecular dynamics (MD) simulations and quantum binding energy calculations, we identified druggable pockets in various conformational states of S1 and all possible binding pockets for cortisol and dexamethasone. Using a novel combination of limited proteolysis and mass spectrometry, we discovered cortisol binding/interaction regions of SARS-CoV-2 S1 which matched multiple interacting residues and druggable pockets we identified through our *in silico* analyses. Through biochemical interaction studies we confirmed that cortisol and dexamethasone bind directly to wild type SARS-CoV-2 S1 (but not ACE2). Moreover, cortisol concentration-dependently inhibits the interaction between ACE2 and the SARS-CoV-2 S1 Beta variant containing mutations E484K, K417N and N501Y. Our data predict that the specific mutations in the highly infectious Delta and Omicron variants of concern may impact the binding of glucocorticoids to S1 which in turn may decrease glucocorticoids inhibition of S1-ACE2 interactions. Our findings suggest that the different interactions of cortisol and dexamethasone with SARS-CoV-2 S1 and variants of concern, including Delta and Omicron, may provide a form of differential innate protection – a subject that warrants further research.

## Methods

### Mapping Druggable Pockets on SARS-CoV-2 S1

The SARS-CoV-2 spike glycoprotein trimer (PDB: 6VYB) was used for the study. The experimental cryo-EM structure has missing regions that are built-in using SWISS-MODEL (20). MD simulations are performed to allow the structure (PDB: 6VYB) to relax to a more favorable conformation (21). The 10 µs simulation trajectory was obtained from D. E. Shaw Research (22). The long timescales and overlapping conformations provide confidence for further structural investigation for biologically relevant events. As no major conformational change was observed, the last structure in the trajectory was used as the search model. The druggable pockets were identified using the Fpocket platform (23, 24). All the pockets below a 9 Å cut off were discarded as they would not fit cortisol or dexamethasone. Each pocket was named as STP (SiTePoint) followed by a number as per Fpocket nomenclature. Each of the selected pockets was docked with each ligand using AutoDockVina (v 1.0.2) to capture the potential ligand binding poses (25). The structures of ligands (i.e., cortisol and dexamethasone) were downloaded from the PDB. The ligand was prepared for Vina using AutoDockTools version 1.5.4 where Gasteiger charges and polar hydrogens were added (26). The parameter “Exhaustiveness” which determines how comprehensively the program searches for the lowest energy conformer, was set to the default value (eight). The S1 trimer-ligand complex in the top-ranking docking pose per pocket was used for MD simulations. We refer to cortisol and dexamethasone as “HCY” and “DEX” respectively in the figures pertaining to their structure (**Supplementary Figure 2**).

### Molecular Dynamics

To relax the ligand-bound structure after docking and obtain the most favorable pose of the ligand in a given pocket, MD studies were performed. The simulation studies of S1 trimer bound to each ligand in a specific pocket were performed using graphics processing unit (GPUs) accelerated Assisted Model Building with Energy Refinement (AMBER) suite version 18 (27) using Amber ff14SB force field (28). The glycan chains were removed for the simulation studies and the asparagine residues were kept intact. The force fields for cortisol and dexamethasone were prepared using the PyRED server (29). In each S1 trimer- ligand complex, hydrogen atoms were positioned using the tleap module. The protein was centered in a truncated octahedral box solvated with the OPC water molecule (30) having a 10 Å cut off in all directions. Overall neutrality of the system was maintained by the addition of counterions. The systems were minimized using a two-phase energy minimization procedure, which included 2500 cycles of steepest descent and 2500 cycles of a conjugate gradient with solute atoms restrained by a harmonic potential with a force constant of 1 kcal mol−1 Å (2). This was followed by 5000 steps of unrestrained whole system minimization. Density equilibration with weak harmonic restraints [1 kcal mol−1 Å (2)] on the solute molecule was performed for 125 picoseconds followed by unrestrained equilibration for 500 picoseconds under constant-pressure and constant-temperature conditions. All simulations ran with constraints using the SHAKE algorithm on hydrogen-linked bonds (31). Langevin dynamics were used to maintain a constant temperature of 300 K throughout the simulations. Finally, 10 nanosecond MD simulations were performed using an NPT ensemble without restraints. Visual Molecular Dynamics was used for visualizing the trajectories after simulations (32). Ligplot was used to map the hydrogen and hydrophobic bonding patterns between the ligand and the residues from the S1 trimer (33). All the three-dimensional representations were generated using PyMOL (34).

### Binding Energy Calculations

Molecular mechanics generalized Born surface area (MM/GBSA) calculation were performed on the ligand-bound S1 to obtain the binding free energy (35, 36). These calculations are more accurate compared to the binding free energy obtained after molecular docking. It has been reported that 4ns simulations are enough to perform MM/GBSA calculations. In the presented study, the last 10 frames of the 10th nanosecond after simulations were used for binding energy calculation. The high affinity pockets (binding affinity >13 kcal/mol) are selected for crafting figures for the current study.

### Complementary and Redundant Biochemical Approaches to Assess the Interactions Between Glucocorticoids and SARS-CoV-2 S1 Spike Protein

For all assays, the effects of cortisol and dexamethasone on S1 and S1/ACE2 interactions were compared to those of their vehicle (a diluted ethanolic solution in phosphate saline solution, PBS). Stock solutions were prepared by dissolving cortisol and dexamethasone in absolute ethanol. Working solutions of cortisol and dexamethasone or vehicle contained a ratio of ethanol to PBS of 1:10 (5), 1:10 (4), 1:10 (7) or 1:10 (8) (v/v).

*Identification of cortisol binding peptides by limited proteolysis-coupled LC-MS to detect cortisol binding sites on SARS-CoV-2 S*: To identify cortisol binding sites on SARS-CoV-2 S1 protein, we applied a recently developed proteomics approach that allows identification of small ligand binding sites on a proteome-wide scale (37, 38). The protein of interest is mixed with the putative ligand and any consequential perturbations/conformational changes of protein structure are revealed by double-protease digestion (first, with a nonspecific protease under native conditions and next with trypsin under denaturing conditions) (37, 38). We incubated 0.1 µg/µL SARS-CoV-2 S1 (Catalog# RKNCOVS1H, Reprokine, USA) with 1 µM cortisol or vehicle for 30 minutes followed by addition of 0.001 µg/µL of thermolysin (a non-specific metalloproteinase, Catalog#T7902, Sigma Aldrich, USA). After 30 minutes at 37°C, the proteolysis reaction was stopped by adding 0.5 µM O-phenanthroline and freezing on dry ice. Next, a proteomics workflow involving shotgun was applied to measure ligand perturbation-dependent proteolytic patterns in the samples to identify SARS-CoV-2 S1 peptides that were either protected or proteolyzed due to the structural perturbations induced by cortisol, compared to vehicle. The resulting peptides were digested with trypsin and characterized by mass spectrometry. Briefly, samples were reduced (200 mM DTT in 50 mM ammonium bicarbonate), alkylated (200 mM iodoacetamide in 50 mM bicarbonate), digested with trypsin overnight at 37°C, and dried under vacuum. The dried samples were reconstituted in 4% acetonitrile, 0.2% formic acid and then zip-tipped. The tryptic peptides were resolved and ionized by using nano flow HPLC (Easy-nLC 1000, Thermo Scientific) coupled to a Q Exactive Orbitrap mass spectrometer (Thermo Fisher Scientific) with an EASY-Spray capillary HPLC column (ES800A, 75um x 15cm, 100Å, 3μm, Thermo Scientific). The mass spectrometer was operated in data-dependent acquisition mode, recording high-accuracy and high-resolution survey orbitrap spectra using external mass calibration, with a resolution of 35,000 and m/z range of 300–1700. The twelve most intense multiply charged ions were sequentially fragmented by using higher-energy C-trap dissociation (HCD), and spectra of their fragments were recorded in the orbitrap at a resolution of 17,500; after fragmentation, all precursors selected for dissociation were dynamically excluded for 30s. Data was processed using Proteome Discoverer 1.4 (Thermo Fisher Scientific) and the database was searched using SEQUEST (Thermo Fisher Scientific). Search parameters included a strict false discovery rate (FDR) of 0.01, a relaxed FDR of 0.05, a precursor mass tolerance of 10 ppm and a fragment mass tolerance of 0.01 Da. Peptides were searched with carbamidomethyl cysteine as a static modification and oxidized methionine and deamidated glutamine and asparagine as dynamic modifications.

*Application of cortisol-Acetylcholinesterase conjugate assay to confirm the direct interaction between cortisol and SARS-CoV-2 S1:* We incubated SARS-CoV-2 S1 (Catalog# RKNCOVS1H, Reprokine, USA) with cortisol at a molar ratio of 2:1 (S1: cortisol) for 1 hour. The mixtures were centrifuged (10 minutes at 10000 x g) in spin filter (30 kDa cut-off). The flowthrough, containing unbound free cortisol, was collected and the amount of cortisol was measured using Cortisol Express ELISA kit (Catalog#500370, Cayman Chemicals, USA). This kit works based on competition between cortisol in the sample and a cortisol-Acetylcholinesterase conjugate for binding to anti-cortisol antibody. A fluorescent substrate was used to measure the quantity of the cortisol-Acetylcholinesterase conjugate and the % binding of the cortisol-Acetylcholinesterase conjugate to the anti-cortisol antibody was calculated relative to maximum binding (negative control with no free cortisol). The quantity of cortisol in the sample is inversely proportional to the fluorescence signal and was calculated by subtracting the % binding of the cortisol-Acetylcholinesterase conjugate to the anti-cortisol antibody from 100%.

*Application of protein thermal stability assays to confirm the direct interaction between cortisol and SARS-CoV-2 S1*: Two complementary thermal stability assays were performed:

Assay 1: The GloMelt™ Thermal Shift Protein Stability Kit (Catalog#33021-T, Biotium, USA) was used to determine any increase or decrease in thermal stability of SARS-CoV-2 S1 protein (Catalog#Z03501-1, GeneScript, USA) (by monitoring the increase in fluorescence which represents unfolding of the protein) in the presence or absence of cortisol or dexamethasone. SARS-CoV-2 S1 (0.5 mg/mL; 3.85 µM) was mixed with increasing concentrations of cortisol or dexamethasone (100 nM, 1 µM, 10 µM) or vehicle and 1x dye (provided with the kit) made up to a total volume of 20 µL using the diluent provided with the kit. The reaction mixtures were heated from 25°C to 99°C at a rate of 0.05°C per second and fluorescence was measured using Roche LightCycler^®^ 480 Instrument II at Ex/Em = 470/510 nm. Experiments were conducted at least in duplicates following the standard protocol provided by the manufacturer.

Assay 2: Thermal stability of SARS-CoV-2 S1 (Catalog#Z03501-1, GeneScript, USA) was assessed in the presence or absence of increasing concentrations of cortisol by heating the mixture followed by detection of SARS-CoV-2 S1 protein by SDS-PAGE. SARS-CoV-2 S1 0.762 µM (0.1 mg/mL) was mixed with cortisol or vehicle at a molar ratio of 1:10 or 1:100 (S1: cortisol). The mixtures were then heated at increasing temperatures (37°C, 50°C, 60°C, 70°C, 80°C, 85°C and 90°C) for 10 minutes at each temperature. Between each temperature, the mixture was centrifuged at 20000 x g for 2 minutes and 10 µL of the supernatant was collected and mixed with equal volume of a reducing sample buffer (150 mM Tris-HCL, pH 6.8, 15% (w/v) SDS, 30% (v/v) glycerol and 10% (v/v) 2-mercaptoethanol), and subjected to electrophoresis on a 10% SDS-PAGE gel. The protein bands were detected using Zn-Imidazole reverse stain technique (39, 40).The experiment was repeated to compare the thermal stability of SARS-CoV-2 S1 at 85°C relative to 37°C in the absence and presence of cortisol. All the steps remained the same except for the mixture being heated at only 37°C followed by heating at 85°C.

### Fluorescent Activity Assay to Test Whether Corticosteroids and ACE-2 Interact

The activity of recombinant ACE2 (Catalog# P1535, BioVision, USA) was measured as rate of increase in fluorescence using the ACE2 Activity Assay Kit (Fluorometric) (Catalog#ab273297, Abcam, UK) in the presence or absence of SARS-CoV-2 S1 (Catalog# RKNCOVS1H, Reprokine, USA), cortisol and/or dexamethasone. Recombinant ACE2 (Catalog# ab273687, Abcam, UK) at a final concentration of 0.0005 µg/µL (74.4 pM) was mixed with increasing concentrations of SARS-CoV-2 S1 (0.025 µg/µL (192nM), 0.05 µg/µL (385 nM), 0.1 µg/µL (769 nM)) or cortisol (10nM, 100nM) or dexamethasone (10nM, 100nM) or a mixture of cortisol and dexamethasone at a 1:1 ratio (10nM, 100nM) or ACE2 inhibitor (5 mM) or ethanol (vehicle). The fluorescent substrate was added (1x) and the total reaction mixture was made up to 80 µL using the provided assay diluent. Fluorescence was measured at Ex/Em = 320/420 nm at a constant temperature of 25°C at 1 min intervals for up to 1 hour.

### Assessment of Inhibition of S1-ACE2 Interaction by Corticosteroid Compounds and Anti-SARS-CoV-2 S1 Antibody

SARS-CoV-2 S1 Protein-ACE2 Binding Inhibitor Screening Kit (Catalog# K2050, BioVision, USA) was used to test the inhibition of S1-ACE2 interaction by cortisol (Catalog# H4001, Sigma Aldrich, USA) and dexamethasone (Catalog# D1756, Sigma Aldrich, USA) individually or in combination in the presence or absence of a Hu chimera anti-SARS-CoV-2 S1 monoclonal antibody (Catalog# A02038, GeneScript, USA). In this assay, the binding of immobilized SARS-CoV-2 S1 protein to biotinylated human ACE2 is detected using Streptavidin-HRP which subsequently reacts with a 3,3’,5,5’-Tetramethylbenzidine (TMB) substrate generating a blue colored product that changes to yellow when the stop solution is added, and absorbance is measured at 450 nm. Cortisol and Dexamethasone solutions were prepared by first dissolving in ethanol (vehicle) which was then diluted with the assay diluent (Catalog# K2050-100-8, Abcam, UK) to 10 mM to prepare stock solutions. Concentrations of cortisol and dexamethasone tested were 1 nM, 10 nM, 100 nM and 1000 nM either individually or in combination. Concentrations of anti-SARS-CoV-2 S1 antibody tested were 10 nM and 100 nM. Experiments were conducted at least in duplicates following the standard protocol provided by the manufacturer.

The effect of cortisol on the interaction between ACE2 and SARS-CoV-2 S1 Beta variant (E484K, K417N, N501Y) was determined using a similar adapted interaction inhibition assay. SARS-CoV-2 S1 Beta variant (Catalog#Z03631-100, GeneScript, USA) (4 µg) was pipetted into the wells of a Pierce™ Nickel Coated Plate (Catalog#15142, Thermo Scientific, USA) and incubated at 23°C for 2 hours to immobilize S1 to the wells. After washing (three times) with 200 µL of the 1X wash buffer (Catalog# K2050, BioVision, USA), increasing concentration of cortisol (0 – 1000 nM) was added to the different wells and incubated for 30 minutes. Recombinant ACE-2 protein Fc chimera (Catalog#ab273687, Abcam, UK) (100 µL; final concentration 0.01 µg/µL) was added to the wells and incubated for 1 hour. The wells were washed four times with 200 µL of the 1X wash buffer and 100 µL of a goat anti-Fc antibody HRP conjugate (Catalog#ab97225, Abcam, UK) (final concentration 0.015 µg/µL) was added and incubated for 1 hour. The wells were washed four times with 200 µL of the wash buffer and developed using the 3,3’,5,5’-Tetramethylbenzidine (TMB) substrate (Catalog# K2050, BioVision, USA) as described above and fluorescence was measured at 450 nm.

### Statistical Analysis

SigmaPlot 14 (Systat Software, San Jose, CA) was used to conduct statistical analysis on the results and plot graphs. One way ANOVA was performed, where appropriate (indicated in the figure legends), to determine statistical significance in the difference between conditions. For all experiments, the n value presented in the figure legends refer to distinct and independent sample measurements. Data are presented as mean ± standard error of mean.

## Results

### Cortisol and Dexamethasone Interact With SARS-CoV-2 S1 in Multiple Pockets Identified by Simulations and Quantum Mechanical Binding Energy Calculations

The SARS-CoV-2 S1 trimer is dynamic, with the three RBD domains being more dynamic than the NTD domains (**Supplementary Figure 3**). In the open conformation (PDB: 6VYB), the RBD domains of each chain make different contacts with other RBD domains and the NTD domains of different chains. These interdomain interfaces are predicted to bear plausible pockets for cortisol or dexamethasone, which vary in their binding affinity. Multiple plausible binding pockets suggest that these ligands can bind to spike S1 and lock the trimer in certain conformations. Such locked conformations could impede the interactions of spike S1 with the ACE2 receptor.

The MD simulated structure of S1 protein in its relaxed open conformation was subjected to F-pocket search analysis, which identified 162 druggable pockets. AutoDock Vina helped to dock cortisol or dexamethasone into each of these pockets. The binding energy value from docking as well as visual analysis of all the above pockets helped identify real pockets versus transient pockets. Transient pockets were the ones where one or two residues of the protein were in 4 angstrom radius of the ligand suggesting weak interaction interface. Thus, the number of pockets were narrowed down to 52. This group of pockets had the minimum dimensions to position cortisol or dexamethasone and had multiple residues of S1 interacting with the ligand, suggestive of a more stable interaction. Cortisol and dexamethasone have similar dimensions (**Supplementary Figure 2**) and each of the 52 pockets was accessed for binding with both ligands.

The stability of the S1- ligand complex, when the ligands were placed in each of the 52 chosen pockets, where further subjected to an unrestrained MD simulation for 10 nanoseconds. These final simulations helped the side chains of the residues in the pockets to adjust and form the energetically feasible bonds with the ligand. Thus, the energetically minimized S1-ligand complex was captured in the most favourable conformation where the particular ligand interaction is feasible in the chosen pocket. The pockets were located on the NTD, RBD, NTD-RBD interface, and the RBD-RBD interface. The binding affinity of each ligand in the selected pockets was accessed using the MM/GBSA calculations. The trend of the binding affinity values associated with each pocket, for both ligands, was highly reliable as the energetically minimized S1-ligand complex structure was used for the calculations.

**Figure 1:**
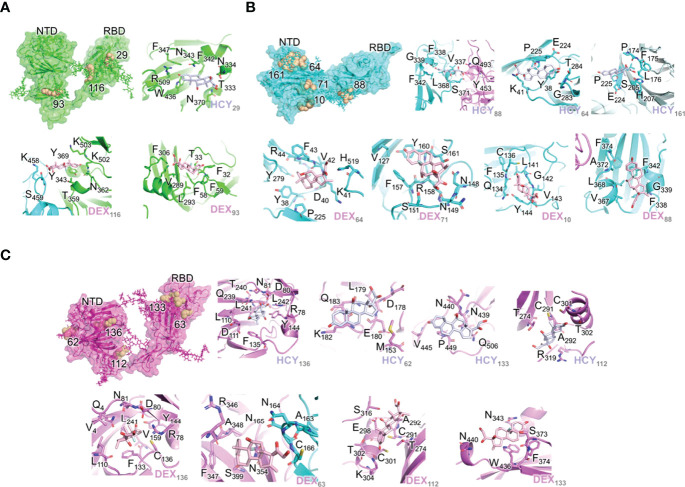
Interactions of the ligand/pocket pairs in chain A (green), chain B (cyan) and chain C (magenta) of the S1 protein**. (A)** The pockets on each chain A were ranked according to the binding affinity. The pockets where the ligands are predicted to bind with a higher affinity (>13 kcal/mol) after MD simulation are shown (**Table 1**). From left to right: High affinity binding pockets for cortisol and dexamethasone; Cortisol pocket 29; Dexamethasone pocket 116; Dexamethasone pocket 93. **(B)** The pockets on chain B were ranked according to the binding affinity. The pockets where the ligands are predicted to bind with a higher affinity (>13 kcal/mol) after MD simulation are shown (**Table 3**). From left to right: High affinity binding pockets for cortisol and dexamethasone; Cortisol pocket 88; Cortisol pocket 64; Cortisol pocket 161; Dexamethasone pocket 64; Dexamethasone pocket 71; Dexamethasone pocket 10. Dexamethasone pocket 88. **(C)** The pockets on chain C were ranked according to the binding affinity. The pockets where the ligands are predicted to bind with a higher affinity (>13 kcal/mol) after MD simulation are shown (**Table 2**). From left to right: High affinity binding pockets for cortisol and dexamethasone; Cortisol pocket 136; Cortisol pocket 62; Cortisol pocket 133; Cortisol pocket 112; Dexamethasone pocket 136; Dexamethasone pocket 63; Dexamethasone pocket 112; Dexamethasone pocket 133. The ligand and pocket ID have been shown in the bottom right-hand corner of each image.

As the three S1 monomers of the trimeric assembly have slightly different orientations (**Supplementary Figure 3** and **Supplemental Movie**), the pockets identified were grouped under each chain (**Tables 1**–**3**, **Figures 1A–C).** The binding affinity of the chosen pockets improved after energy minimizations compared to that obtained after docking. The change of trend of binding energies calculated from docking versus simulations is demonstrated in **Tables 1**–**3**. For chain A monomer of the spike trimer, nine chosen pockets were subjected to MD based energy minimization followed by MM/GBSA calculations. To delineate the residue level interactions, only the pockets displaying the highest affinity with the respective ligands were chosen for figure display, where a cut-off of 13 kcal/mol was used. This cut off was set to limit the number of figure panels per chain, for the readers. In our analysis each of the binding pockets were observed to interact with cortisol and dexamethasone with varying affinity. This is expected in biological scenario, and thus provides confidence to our in-silico analysis pipeline and the outcomes presented in this article. The pocket, STP_29, located in the RBD domain of chain A has highest affinity to cortisol (**Figure 1A**) compared to other cortisol binding sites on chain A (**Table 1**). This pocket ranks comparatively lower in its ability to interact with dexamethasone (**Table 1**). Dexamethasone, on the other hand, binds tighter to STP_116 located on the RBD domain (**Figure 1A**). STP_116 shows a comparatively lower affinity to cortisol (**Table 1**). Our analysis showed that the second ranking high affinity binding pockets were located on the NTD (**Figure 1A** **;** **Table 1**). Although there is structural similarity between cortisol and dexamethasone, the differences in the side chains necessitates additional unique interactions which contribute to the increase in binding affinity and hence the specificity of the site for one of the two ligands.

**Table 1:** Binding energies of cortisol and dexamethasone for each S1 pocket of chain A after docking and MD.

	Cortisol					Dexamethasone		
Pocket ID	Docking	Simulation			Pocket ID	Docking	Simulation	
		ΔG	Std. Dev.				ΔG	Std. Dev.
**STP_29**	-6.0	-16.8	1.4	**STP_116**	-5.3	-16.1	4.2
**STP_92**	-5.9	-12.8	2.1	**STP_93**	-6.0	-14.2	2.9
**STP_93**	-6.0	-12.0	1.7	**STP_103**	-6.6	-11.3	2.0
**STP_120**	-6.4	-11.1	1.5	**STP_84**	-6.2	-11.3	2.1
**STP_87**	-5.3	-9.8	2.8	**STP_120**	-6.9	-8.2	3.0
**STP_4**	-6.2	-9.6	1.9	**STP_87**	-4.8	-5.5	1.9
**STP_116**	-5.7	-7.3	2.7	**STP_92**	-6.5	-2.6	2.4
**STP_103**	-6.4	-2.9	0.9	**STP_4**	-5.9	-2.3	1.6
**STP_84**	-5.9	-2.2	3.6	**STP_29**	-5.7	0.7	0.4

The binding energy is shown as kcal/mole and the values are arranged in a descending order. Pockets are color coded (NTD- blue and RBD- green). A higher negative free energy (ΔG) is an indication of higher affinity and a stable complex. The affinity measurements obtained from docking calculations are shown for comparison with that obtained after simulations. This indicates the stabilization of protein-ligand complex after simulation.

**Figure 2:**
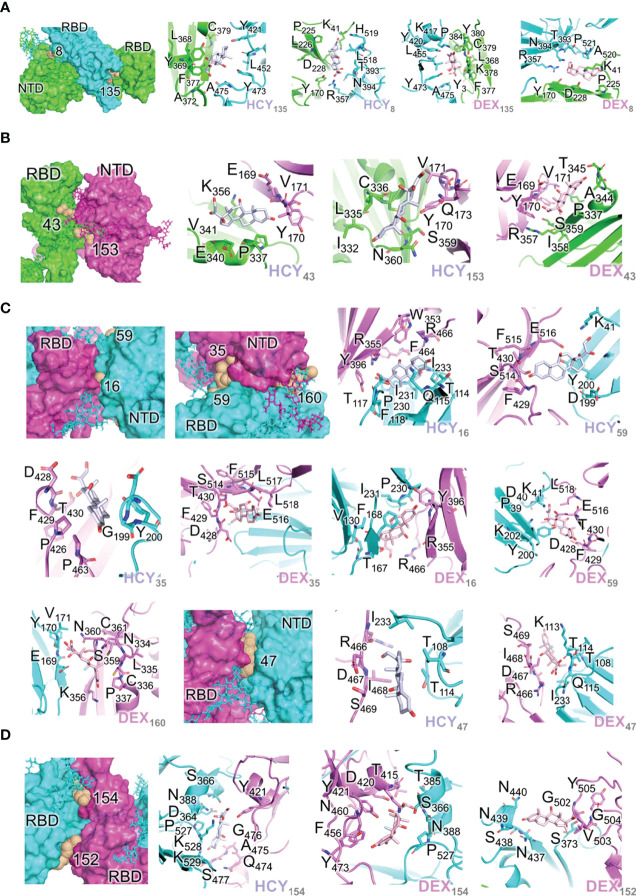
Interactions of the ligand/pocket pairs on the interfaces between chains A, B and C** (A)** Interactions of the ligand/pocket pairs on the interfaces between chain A (green) and chain B (cyan) of the S1 protein (**Table 4**). From left to right: High affinity binding pockets for cortisol and dexamethasone identified on the interface; Cortisol pocket 135; Cortisol pocket 8; Dexamethasone pocket 135; Dexamethasone pocket 8. **(B)** Interactions of the ligand/pocket pairs on the interfaces between chain A (green) and chain C (magenta) of the S1 protein (**Table 4**). From left to right: High affinity binding pockets for cortisol and dexamethasone identified on the interface; Cortisol pocket 43; Cortisol pocket 153; Dexamethasone pocket 43. **(C)** Interactions of the ligand/pocket pairs on the interfaces between chain B (cyan) and chain C (magenta) as shown in **Table 4**. From left to right: High affinity binding pockets for cortisol and dexamethasone identified on the interface; High affinity pockets identified between NTD of chain C and RBD of chain B; Cortisol pocket 16; Cortisol pocket 59; Cortisol pocket 35; Dexamethasone pocket 35; Dexamethasone pocket 16; Dexamethasone pocket 59; Dexamethasone pocket 160; High affinity pocket 47 identified between the interface of NTD of chain B and RBD of chain C; Cortisol pocket 47; Dexamethasone pocket 47. **(D)** Interactions of the ligand/pocket pairs on the interfaces between RBD of chain B (cyan) and RBD of chain C (magenta). The binding energies are shown in **Table 4**. The pockets where the ligands are predicted to bind with a higher affinity (>13 kcal/mole) after MD simulation are shown (**Table 4**). From left to right: High affinity binding pockets for cortisol and dexamethasone identified on the interface; Cortisol pocket 154; Dexamethasone pocket 154; Dexamethasone pocket 152.

Similarly, for chain B monomer, 14 ligand binding pockets were identified. As mentioned above the structures of chain A and chain B were not identical (**Supplementary Figure 3A**). This conformational plasticity allowed the formation of new high affinity binding pockets in chain B, which were not observed in chain A, such as STP_64, STP_161, STP_71. The pockets showing highest affinity to cortisol and dexamethasone are STP_88, located on the RBD domain (**Figure 1B** **;** **Table 2**) and STP_64, located on the NTD domain (**Figure 2B** **;** **Table 2**). STP_64 does bind to cortisol with high affinity (**Table 2**). Twelve pockets were observed in chain C monomer (**Table 3**). Interestingly, the pocket, STP_136, located on the NTD, showed the highest binding energy values for both cortisol and dexamethasone (**Table 3**; **Figure 1C**). While, for dexamethasone, the second high affinity pocket is located on the RBD (STP_63; **Figure 1C**); for cortisol it is located on the NTD (STP_62; **Figure 1C**) (**Table 3**). STP_62 had comparatively lower affinity for dexamethasone (**Table 3**).

**Table 2:** Binding energies of cortisol and dexamethasone for each S1 pocket of chain B after docking and MD.

	Cortisol					Dexamethasone
Pocket ID	Docking	Simulation			Pocket ID	Docking	Simulation	
		ΔG	Std. Dev.				ΔG	Std. Dev.
**STP_88**	-6.3	-17.0	3.2	**STP_64**	-8.7	-22.3	3.7
**STP_64**	-8.3	-15.1	1.9	**STP_71**	-8.0	-16.6	2.2
**STP_161**	-8.5	-14.4	2.6	**STP_10**	-6.0	-15.0	1.4
**STP_155**	-6.8	-11.8	1.9	**STP_88**	-6.8	-13.7	1.4
**STP_143**	-8.3	-9.9	3.0	**STP_90**	-7.3	-12.7	3.0
**STP_90**	-7.0	-8.6	1.4	**STP_161**	-8.8	-10.3	1.2
**STP_158**	-7.6	-7.9	2.2	**STP_143**	-8.2	-9.4	4.3
**STP_10**	-5.5	-5.4	1.5	**STP_138**	-6.0	-7.6	1.1
**STP_138**	-5.7	-1.2	2.3	**STP_155**	-6.2	-6.4	2.8
**STP_71**	-7.7	ND	ND	**STP_158**	-7.1	-3.1	1.8
**STP_3**	-1.9	ND	ND	**STP_3**	3.6	ND	ND
**STP_82**	-5.5	ND	ND	**STP_134**	-4.9	ND	ND
**STP_52**	-7.6	ND	ND	**STP_52**	-7.6	ND	ND
**STP_134**	-5.2	ND	ND	**STP_82**	-4.6	ND	ND

The binding energy is shown as kcal/mole and the values are arranged in a descending order. Pockets are color coded (NTD- blue and RBD- green). Values < 0.1 are not defined (ND).

**Table 3:** Binding energies of cortisol and dexamethasone for each S1 pocket of chain C after docking and MD.

	Cortisol					Dexamethasone
Pocket ID	Docking	Simulation			Pocket ID	Docking	Simulation	
		ΔG	Std. Dev.				ΔG	Std. Dev.
**STP_136**	-7.7	-34.9	3.0	**STP_136**	-6.7	-24.2	2.2
**STP_62**	-7.1	-17.8	2.2	**STP_63**	-6.7	-23.3	3.4
**STP_133**	-7.6	-14.2	1.9	**STP_112**	-6.4	15.5	2.2
**STP_112**	-6.6	-13.0	1.7	**STP_133**	-7.4	13.8	1.7
**STP_44**	-6.6	-11.2	1.8	**STP_124**	-5.9	-14.4	2.5
**STP_53**	-4.8	-8.5	1.7	**STP_114**	-7.2	11.4	1.7
**STP_114**	-7.3	-7.3	1.4	**STP_53**	-4.8	-6.9	2.5
**STP_63**	-6.5	-5.7	2.6	**STP_36**	-6.1	-5.4	1.4
**STP_20**	-6.0	-4.9	3.4	**STP_44**	-6.5	-5.2	2.8
**STP_51**	-7.2	-4.4	1.5	**STP_20**	-5.4	-4.6	1.7
**STP_36**	-5.9	0.3	0.2	**STP_62**	-6.9	-1.0	2.2
**STP_124**	-5.9	ND	ND	**STP_51**	-6.6	0.5	0.4

The binding energy is shown as kcal/mole and the values are arranged in a descending order. Pockets are color coded (NTD- blue and RBD- green).

As the three monomers make contacts among each other to form the spike S1 trimer, there are 17 pockets identified in these interfaces that have propensity to interact with the two ligands independently. These pockets were hence grouped separately (**Table 4**; **Figures 2A–D**). Although the domains of the three chains are identical, unique pockets are identified between two unique chains which may not be observed when the third chain was introduced into the equation. This was because of the structural dynamics and plasticity of the individual spike monomers as a part of spike S1 trimer (**Supplementary Figure 3**). As expected, pockets were found between NTD of one monomer and RBD of a second monomer, and also between RBDs of different monomers (**Table 4**; **Figure 2**). As the NTDs of monomers do not make any contacts with each other in spike S1, no pockets could be expected. The two ligands can bind to the pocket STP_135 and STP_8, located at the interface between chain A and chain B, with high affinity (**Table 4**; **Figure 2A**). The interface between chains A and C could interact with high affinity with both ligands at the pocket STP_43 (**Table 4**; **Figure 2B**). The interface between chain B (NTD) and chain C (RBD) harbours a pocket STP_47 (**Table 4**; **Figure 2C**), which has the propensity to interact with both cortisol and dexamethasone with similar affinity. The two pockets, STP_154 and STP_152, identified in the interface between the RBDs of chains B and C, interact with the two ligands (**Table 2**; **Figure 2D**). Taken together, we identified unique high affinity binding sites in each chain and at the interface regions between various domains that are created by the virtue of their non-identical conformations and protein dynamics captured *via* simulation studies, which can mimic biological scenario.

**Table 4:** Binding energies of cortisol and dexamethasone for each S1 pocket at the interfaces after docking and MD.

	Cortisol					Dexamethasone
Pocket ID	Docking	Simulation			Pocket ID	Docking	Simulation	
		ΔG	Std. Dev.				ΔG	Std. Dev.
**NTD of Chain A and RBD of chain B**				**NTD** of Chain A and **RBD** of Chain B			
**STP_135**	-8.4	-25.5	4.0	STP_135	-7.8	-30.2	2.5
**STP_8**	-6.6	-23.1	3.1	STP_8	-6.8	-22.0	3.3
**STP_157**	-5.9	-12.7	2.2	STP_162	-8.1	-2.7	2.4
**STP_162**	-7.3	-3.9	3.2	STP_157	-5.7	-1.6	1.6
	
**RBD of Chain A and NTD of Chain C**				**RBD** of Chain A and **NTD** of Chain C			
**STP_43**	-7.3	-13.8	1.8	STP_43	-7.2	-24.4	3.6
**STP_153**	-6.9	-13.5	3.0	STP_128	-7.0	-9.7	2.0
**STP_125**	-5.7	-10.8	2.3	STP_125	-5.8	-6.9	2.8
**STP_128**	-6.6	-9.6	2.3	STP_60	-5.5	-6.9	3.1
**STP_156**	-5.5	-6.8	2.6	STP_156	-5.6	-5.3	1.7
**STP_60**	-6.2	0.9	0.4	STP_153	-7.2	-2.0	0.6
	
**NTD of Chain B and RBD of chain C**				**NTD** of Chain B and **RBD** of chain C			
**STP_16**	-3.5	-37.6	3.5	STP_35	-6.6	-26.7	3.1
**STP_59**	-6.9	-18.8	2.6	STP_16	-2.6	-26.3	2.2
**STP_35**	-6.5	-18.4	2.9	STP_59	-6.6	-15.3	2.7
**STP_160**	-8.6	-12.7	3.4	STP_160	-7.7	-15.0	3.9
	
**NTD of Chain B and RBD of chain C**				**NTD** of Chain B and **RBD** of chain C			
**STP_47**	-8.7	-17.4	2.9	STP_47	-8.2	-16.0	3.4
	
**RBD of chain B and RBD of chain C**				**RBD** of chain B and **RBD** of chain C			
**STP_154**	-9.1	-20.0	3.2	STP_154	-9.6	-26.4	3.3
**STP_152**	-6.2	-12.8	2.4	STP_152	-7.0	-15.4	3.3
	

The binding energy is shown as kcal/mole and the values are arranged in a descending order. The interface pockets formed between two domains from different chains are grouped.

### Limited Proteolysis-Coupled Mass Spectrometry-Based Identification of Cortisol Binding Sites on SARS-CoV-2 S1 Experimentally Validates *In Silico* Findings

**Figure 3:**
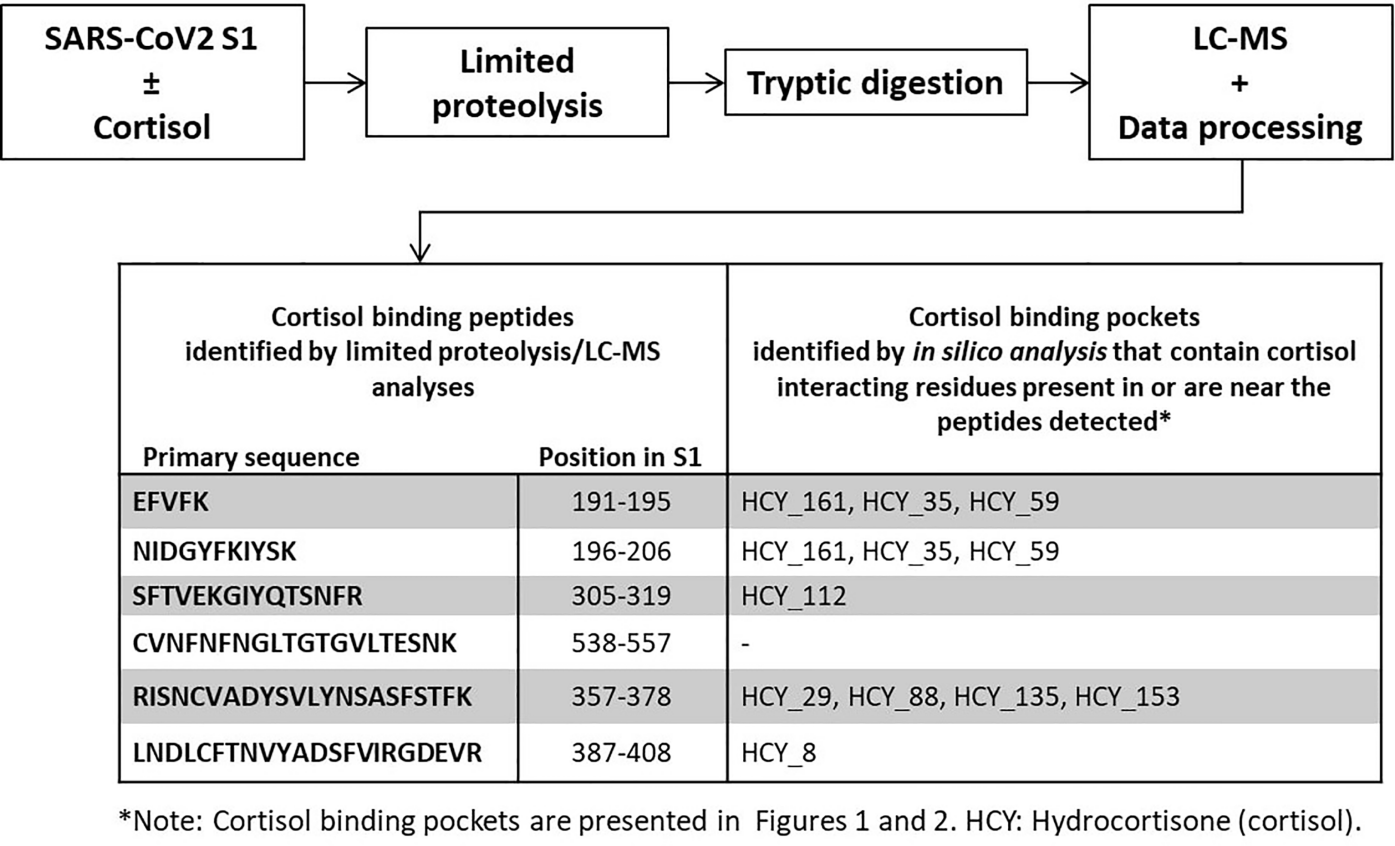
Cortisol binding sites on SARS-CoV-2 S1 identified by limited proteolysis-coupled mass spectrometry. The unique peptides which were detected by mass spectrometry following limited proteolysis of SARS-CoV-2 S1 incubated with vehicle or cortisol represent cortisol binding sites. Full list of detected peptides is shown in **Supplementary Table 1**. Comparison between these cortisol binding sites with the binding pockets we identified through molecular dynamics and binding energy studies reveal multiple common binding sites identified by both methods.

We used a limited proteolysis-coupled mass spectrometry approach to pinpoint binding sites of cortisol on SARS-CoV-2 S1 (37, 38). Binding target identification is based on the principle that small-ligand binding alters (increases or decreases) the protease accessibility of the target protein (37, 38). The binding of the ligand (cortisol) to the target protein (SARS-CoV-2 S1) induces perturbations/conformational changes at the binding sites which can lead to either facilitating or preventing proteolysis by non-specific proteases (thermolysin and trypsin) (37, 38). We subjected SARS-CoV-2 S1 incubated with either cortisol or vehicle to proteolysis by thermolysin followed by trypsin digestion and mass spectrometry-based identification of the resultant peptides (**Supplementary Table 1**). We found that cortisol either facilitated or prevented S1 proteolytic cleavage at discrete sites. Six unique peptides were identified that were only present for SARS-CoV-2 S1 incubated with either cortisol or vehicle (**Figure 3**; **Supplementary Table 1**). Cross-matching of the unique peptides with our above-described *in silico* analyses revealed that these peptides are part of or in the vicinity of cortisol binding pockets identified by our *in silico* studies (**Figures 1** **–** **3**; **Tables 1** **–** **4** and **Supplementary Table 1**).

### Cortisol and Dexamethasone Interact With SARS-CoV-2 S1 and Does Not Affect ACE2 Catalytic Activity

To experimentally confirm whether cortisol directly interacts with SARS-CoV-2 S1, we applied three complementary and redundant biochemical approaches (Methods).

**Figure 4:**
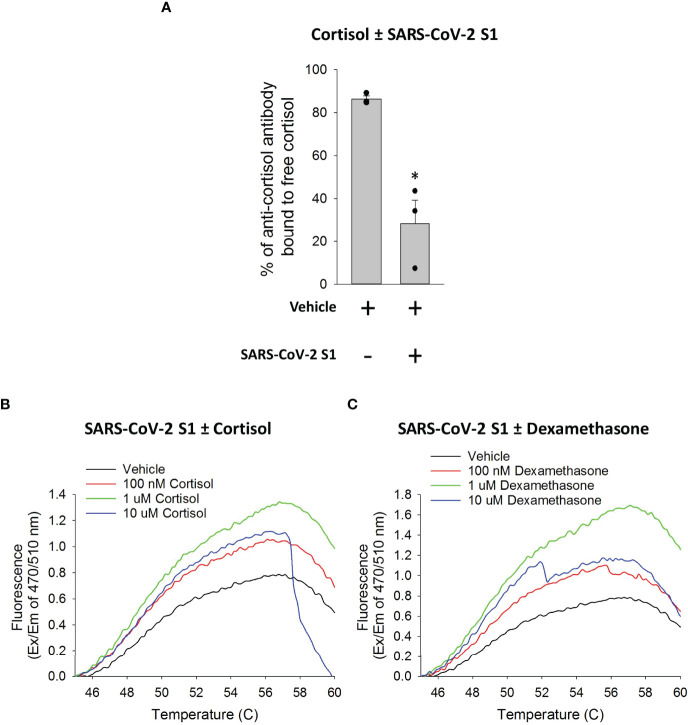
Cortisol directly binds SARS-CoV-2 S1 protein. **(A)** Bar graph showing a decrease in free cortisol in solution in the presence of SARS-CoV-2 S1. Cortisol was incubated with vehicle or SARS-CoV-2 S1 and the sample mixtures were filtered to collect unbound free cortisol in the flow through. The quantity of cortisol in the flow through from the samples was determined as the percentage of anti-cortisol antibody bound to cortisol as opposed to a competitor cortisol-acetylcholinesterase conjugate. Data presented as mean ± standard error (n = 3). *P < 0.05 vs vehicle (1-way ANOVA). **(B, C)** Line plots generated using GloMelt™ Thermal Shift Protein Stability assay showing unfolding (represented by increase in fluorescence) of SARS-CoV-2 S1 at increasing temperatures in the presence or absence of increasing concentrations of cortisol or dexamethasone (100 nM, 1 µM, 10 µM). Data for high concentrations (10 µM) of either cortisol or dexamethasone suggest formations of aggregates in the aqueous conditions of the assay (as indicated by the decay of the signal). Data are representative of two independent experiments.

First, we incubated cortisol with SARS-CoV-2 S1 or vehicle at a molar ratio of 2:1 (S1: cortisol) for 1 hour and measured the residual quantity of free cortisol in the solution using a competitive binding Acetylcholinesterase (AChE) conjugate assay. We found that the presence of SARS-CoV-2 S1 significantly decreased the quantity of free cortisol in the solution, compared to vehicle (**Figure 4A**). These data showed that cortisol directly binds to SARS-CoV-2 S1.

Additional demonstration that both cortisol and dexamethasone directly interact with SARS-CoV-2 S1 was obtained using two different thermal shift protein stability assays (Assays 1 and 2 under Methods). We utilized a fluorescent dye (GloMelt™) that binds hydrophobic residues as a protein unfolds due to increasing temperatures such that an increase in fluorescence reading represents protein unfolding. We heated mixtures of SARS-CoV-2 S1 in the presence or absence of increasing concentrations of cortisol or dexamethasone. The presence of increasing concentrations (< 10 µM) of cortisol or dexamethasone in the solution with SARS-CoV-2 S1 showed a greater increase in fluorescence as temperature increased, compared to vehicle (**Figures 4B, C** **)**. These results showed that cortisol and dexamethasone concentration-dependently facilitated the heat-induced unfolding of SARS-CoV-2 S1, consistent with our hypothesized direct interaction of both cortisol and dexamethasone with SARS-CoV-2 S1. Data for high concentrations (>10 µM) of either cortisol or dexamethasone (**Figures 4B, C** **)** suggested the formation of aggregates in the aqueous conditions of the assay (as indicated by the decay of the fluorescence signal), which precluded the analysis. In the second assay, we heated the solutions of SARS-CoV-2 S1 in the presence or absence of cortisol and measured the fraction of soluble (folded) SARS-CoV-2 S1 remaining as the temperature increased from 37°C to 90°C. The results showed that denaturation/precipitation of SARS-CoV-2 S1 is increased in the presence of increasing concentrations of cortisol (**Supplementary Figure 4A**). Testing the solubility of SARS-CoV-2 S1 at a single temperature point (85°C relative to 37°C) also showed a decrease in SARS-CoV-2 S1 solubility in the presence of cortisol (**Supplementary Figure 4B**).

Together, our biochemical data clearly showed that cortisol and dexamethasone bind to SARS-CoV-2 S1 causing S1 to partially unfold.

To determine whether cortisol and dexamethasone also interact with ACE2, we used an ACE2 activity assay, where ACE2 activity is represented as the rate of increase in fluorescence from fluorophores released when an active ACE2 binds and cleaves a synthetic methoxycoumarin-based peptide substrate. Increasing concentrations of cortisol or dexamethasone did not show any inhibition of ACE2 interaction with its fluorescent substrate (ACE2 activity) (**Supplementary Figure 5A**). As expected, increasing concentrations of SARS-CoV-2 S1 increasingly inhibited ACE2 activity at constant substrate concentration (**Supplementary Figure 5B**), suggesting that SARS-CoV-2 S1 (but neither cortisol nor dexamethasone) competes with the substrate for the same binding sites on ACE2. These data show that glucocorticoids do not bind to the catalytic site in ACE2 nor alter ACE2 conformation in ways that compromise ACE2 enzymatic function.

### Cortisol and Dexamethasone Can Inhibit the Binding of SARS-CoV-2 S1 to ACE2

**Figure 5:**
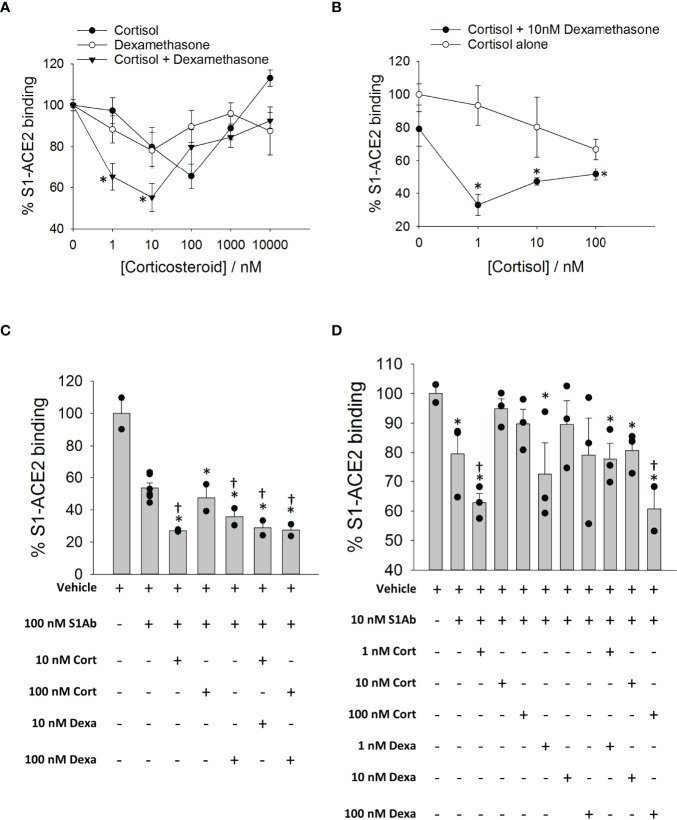
The effects of cortisol and dexamethasone on the binding of SARS-CoV-2 S1 to ACE2. **(A)** Plot showing a dose response on SARS-CoV-2 S1-ACE2 interaction in the presence of increasing concentration of glucocorticoids (cortisol, dexamethasone or a mixture of cortisol and dexamethasone). Each data plot was normalized to vehicle (100% S1-ACE2 binding). Data presented as mean ± standard error (n = 2-6). *P < 0.05 vs cortisol or dexamethasone alone at same concentration (1-way ANOVA). **(B)** Plot showing a dose response on SARS-CoV-2 S1-ACE2 interaction in the presence of increasing concentration of cortisol ± 10 nM dexamethasone. Each data plot was normalized to vehicle (100% S1-ACE2 binding). Each data plot was normalized to vehicle (100% S1-ACE2 binding). Data were measured in at least two independent replicates and are presented as mean ± standard error. *P < 0.05 vs cortisol alone at same concentration (1-way ANOVA). **(C, D)** Bar plot showing the effect of cortisol or dexamethasone on the inhibition of SARS-CoV-2 S1-ACE2 interaction by anti-SARS-CoV-2 S1 antibody (10nM or 100nM). Data are normalized to vehicle (100% S1-ACE2 binding). Data presented as mean ± standard error (n = 2-6). *P < 0.05 vs vehicle (1-way ANOVA); ^†^P < 0.05 vs S1Ab alone (1-way ANOVA).

To determine whether cortisol or dexamethasone can influence the interactions of SARS-CoV-2 S1 with ACE2, we used a solid-phase biochemical interaction assay. We found that cortisol and dexamethasone concentration-dependently inhibit the interaction between SARS-CoV-2 S1 and ACE2, achieving greatest inhibition at 100 nM for cortisol (30% inhibition) and 10 nM for dexamethasone (20% inhibition) relative to vehicle (**Figure 5A**). A combination of cortisol and dexamethasone at a 1:1 molar ratio resulted in significantly greater inhibition at 1 nM and 10 nM relative to cortisol or dexamethasone alone at the same concentrations (**Figure 5A**). Interestingly, at concentrations of 100 nM, 1000 nM and 10000 nM the combination of cortisol and dexamethasone did not show any difference in inhibition compared to cortisol or dexamethasone alone at the same concentrations (**Figure 5A**). Addition of 10 nM dexamethasone to increasing concentrations of cortisol showed consistently greater inhibition across the range of concentrations tested relative to inhibition achieved by cortisol alone (**Figure 5B**). However, the overall pattern of the dose-response plots was a ‘V’ shape as further increasing the concentrations of either cortisol or dexamethasone beyond an optimum concentration resulted in a dramatic decrease of the inhibitory potential of these glucocorticoids. Therefore, the effective concentration of glucocorticoids is limited within a range of concentrations below or above which no inhibition of SARS-CoV-2 S1 interaction with S1 is observed.

Next, we assessed whether cortisol and dexamethasone influence the inhibition of S1/ACE2 interactions by neutralizing S1 antibodies. Cortisol and dexamethasone cooperatively increased the inhibition of interaction between SARS-CoV-2 S1 and ACE2 by a human chimeric monoclonal anti-SARS-CoV-2 S1 antibody (**Figures 5C, D** **)**. Cortisol showed the greatest inhibition when paired with the antibody at a 1:10 (cortisol: antibody) molar ratio when compared to the vehicle or antibody alone (**Figures 5C, D** **)**. The enhanced inhibition was not observed when cortisol was added to the antibody at molar ratios of 1:1 and 10:1 (cortisol: antibody) (**Figures 5C, D** **)**. Dexamethasone alone did not improve the inhibition by the antibody (**Figure 5D**). A mixture of cortisol and dexamethasone at 1:1 molar ratio increased the inhibition by the antibody (100 nM) compared to cortisol or dexamethasone at the concentration of 100 nM (**Figure 5D**). These results showed that ACE2-SARS-CoV-2 S1 interaction may be cooperatively inhibited both by glucocorticoids and cocktails of glucocorticoids and S1 antibodies –a result with clinical translation potential.

Furthermore, we tested whether cortisol is effective in inhibiting the interaction between ACE2 and a mutant SARS-CoV-2 S1 (containing mutations E484K, K417N and N501Y) from a SARS-CoV-2 variant of concern (Beta variant). Cortisol concentration-dependently inhibited the ACE2/SARS-CoV-2 S1 interaction with the most effective cortisol concentration being 100 nM resulting in an approximately 55% inhibition (**Supplementary Figure 6**).

### Influence of Mutations Specific to Beta, Delta and Omicron Variants of SARS-CoV-2 on Glucocorticoids Interactions With S1

Variants of SARS-CoV-2 have emerged comprising mutations in the regions that are important to S1 function and protein stability (41). One region of interest is the RBD whose receptor binding motif (RBM), residues 438 to 506, mediates key interactions with human ACE2 (42). Based on our HCY/DEX specific pocket search followed by simulation assisted binding energy calculations, we intended to explore and predict whether specific S1 variants would affect glucocorticoid interactions with S1.

The inhibitory effect of cortisol on the interaction between ACE2 and SARS-CoV-2 S1 Beta variant (E484K, K417N, N501Y) was confirmed experimentally using an adapted interaction inhibition assay (**Supplementary Figure 6**).

**Figure 6:**
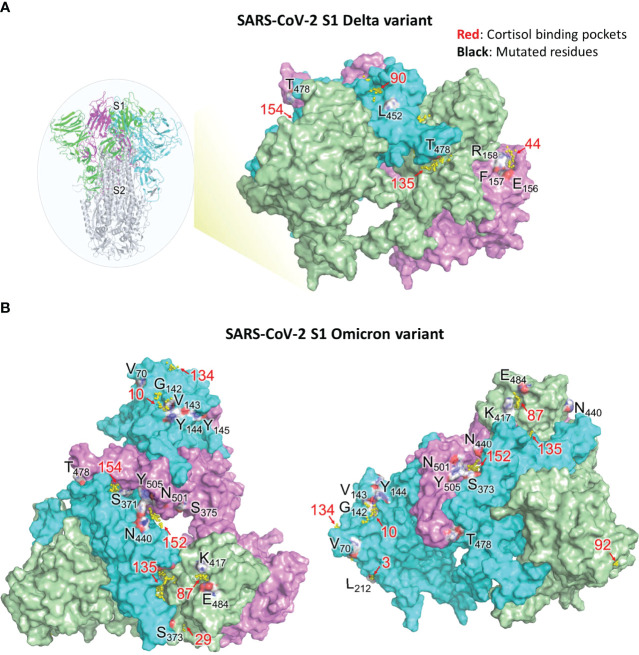
Visual representation of SARS-CoV-2 S1 residues mutated in Delta and Omicron variants and their associated cortisol binding pockets (refer **Tables 5**, **6**). **(A)** Residues of SARS-CoV-2 S1 protein (WT) that are deleted or mutated in the Delta variant along with neighboring glucocorticoid binding pockets. Insert on the left, shows the spike protein of SARS-CoV-2. S1 trimer (three monomers are colored as green, cyan and magenta) and S2 trimer (grey) are shown. The residues, which are not proximal to any binding pocket, are not shown. Pockets (yellow spheres) and their respective numbers are shown. Pocket 154 is buried in between RBD domains of chain B and C The residues are colored based on the element (red for oxygen, blue for nitrogen, white for carbon). **(B)** Residues of SARS-CoV-2 S1 protein (WT) that are deleted or mutated in the Omicron variant along with neighboring glucocorticoid binding pockets. Pockets with their respective numbers are shown as yellow spheres. Chain A (green), chain B (cyan) and chain C (pink) of S1 are shown. Left: Top view of S1 in wild type SARS-CoV-2; Right: Rotated view of S1 focusing on chain B The residues are colored based on the element (red for oxygen, blue for nitrogen, white for carbon). Binding pockets are in red and mutated residues in black.

The location of S1 mutations found in the Delta and Omicron variants were visualized on the structural model using PyMOL. Whether these residues are involved in the formation of the glucocorticoid binding pockets was analyzed. If these residues are involved in pocket formation, then a mutation could impact the ligand binding. If the residues are near the pocket (not a part of the pocket), the propensity of the mutation to affect the secondary structure was taken into consideration (**Tables 5**, **6** **;** **Figures 6A–B**)

**Table 5:** S1 mutations in Delta variant (B.1.617.2) of SARS-CoV-2 and their accessibility to cortisol (HCY) and dexamethasone (DEX) binding.

Deltamutations	Buried residueNot accessible to HCY or DEX	Pocket	Can affect HCY binding	Can affect DEX binding	Nearest residues involved in HCY and/or DEX binding	High affinity pocket
T19R	–	–	–	–		–
E156-	–	STP_44	yes	yes		yes
F157-	–	–	yes	yes	STP_44 (HCY/DEX): E156, R158	yes
R158G		STP_44	yes	yes		yes
L452R	–	–	–	–	STP_90 (HCY/DEX): N450, Y451	yes
T478K	–	–	–	–	STP_135 (HCY/DEX): A475 STP_154 (DEX): Q474	yes
D614G	partly buried	–	–	–	–	–

In cases where the residue is involved in formation of a pocket, the column with header “nearest residue involved in HCY/DEX binding” is not filled to avoid redundancy. Residues in blue font represent residues in the RBM motif.

**Table 6:** S1 mutations in Omicron variant (1.1.529) of SARS-CoV-2 and their accessibility to cortisol (HCY) and dexamethasone (DEX) binding.

S1 mutations in Omicronvariant	Buried residue Not accessible to HCY or DEX	Residue of a pocket	Can affect HCY binding	Can affectDEX binding	Nearest residues involved in HCY/DEX binding as the part of a pocket	High affinity pocket
A67V	yes	–	may affect	may affect	STP_92 (HCY/DEX): W64, F65, H66	yes
H69-	–	STP_134	yes	yes		No
V70-	–		no	no	STP_134 (HCY/DEX): H69	No
T95I		STP_3	yes	yes		No
G142-	–	STP_10	yes	yes		yes
V143-	–	–	may affect	may affect	STP_10 (HCY/DEX): G142, Y144	yes
Y144-	–	STP_10	yes	yes		yes
Y145D	–	–	no	no	STP_10 (HCY/DEX): Y144	yes
N211-	–	STP_82	yes	yes		no
L212I	–	STP_82	yes	yes		no
G339D	–	–	may affect	may affect	STP_43 (HCY/DEX) P337, F338, V341	yes
S371L	–	STP_29	yes	yes		yes
S373P	Interface of two S1 protomers	STP_135 STP_152	yes	yes	STP_29 (HCY/DEX): S371	yes
S375F	–	STP_152	yes	yes		yes
K417N	–	STP_87, STP_135	no	yes	STP_154 (HCY): T415, G416	yes
N440K	–	STP_152	no	yes	STP_29 (HCY): L441	yes
G446S	–	–	–	–		–
S477N	–	STP_154	yes	yes		yes
T478K	–	–	may affect	may affect	STP_154 (HCY/DEX): S477N	yes
E484A	–	STP_87	yes	yes		no
Q493R	–	–	–	–		–
G496S	–	–	–	–		–
Q498R	–	–	–	–		–
N501Y	–	–	–	–	STP_87(HCY): G502 STP_152 (HCY/DEX): G502, V503, G504, Y505	yes
Y505H		STP_152	no	yes		yes
T547K		STP_155,	yes	yes	STP_114 (DEX): L546	yes
D614G	Partially buried	–	–	–	–	–
H655Y	–	–	–	–	–	–

Some residues, deleted or mutated in the Omicron variant, are not identified as a part of the HCY or DEX pockets. For these residues, we have annotated the adjacent residues that are involved in the formation of the nearest HCY/DEX pocket. For the binding energy values of the high affinity sites, refer the **Tables 1**-**4**. If the binding energy of the pocket for one or both ligands is greater than 10 kcal/mol, it is referred in this table as high affinity pocket. Residues in blue fonts are shown to be involved in ACE2 binding in the wild type of SARS-CoV-2. K417 is located outside RBM, however forms salt-bridge based interaction with ACE242.

T19, found in the NTD of S1 (**Supplemental Figure 1**), is buried however the mutation T19R makes it surface-exposed. T19R has no impact on the overall topology of S1 (43), rather stabilizes the neighbouring region by forming 4 hydrogen bonds (instead of one as observed in the wild type). T19R is not present near any pocket and hence is not likely to perturb HCY/DEX interactions on S1. On the contrary, deletion of E158 and R158G mutation led to disruption of 4-5 hydrogen bonds present in the wild type (**Table 5**). The side chain of F157 does not contribute to the formation of STP_44 as it faces away from the pocket. However, its deletion in the Delta variant might perturb the pocket as the adjacent residues E156 and R158 are involved in the formation of the STP_44 (**Table 5**; **Figure 6A**).

Due to the dynamic nature of the S1 RBD, L452 faces the glycan chains on one protomer and is not involved in any glucocorticoid binding pocket formation. L452R is known to stabilize the local environment by formation of 3 hydrogen bonds (43). This residue is located near STP_90 in one of the protomers (**Figure 6A**). The mutation L452R might not affect STP_90 as the side chain of the former faces away from the pocket. Hence the mutation would not affect the binding of HCY or DEX to the pocket.

T478, on the RBD, is located near STP_135 and STP_154 (**Table 5**; **Figure 6A**). However, the side chain faces outwards from the pocket. It is unlikely that T478K would affect the binding of glucocorticoid to this site, which are identified as the high affinity sites between NTD/RBD and RBD/RBD interface respectively (**Table 4**).

D614G has been identified to stabilize the protein, generate high titres of virus *in vitro*, infectivity and is also susceptible to neutralisation (44–46). Molecular dynamics studies have shown that this mutation improves the stability of the spike protein. D614 located in the linker region, is not involved in any pockets presented in this study.

Omicron mutations S371L and S373P located in the RBD domain of chain A are part of the pocket STP_29 (**Table 6**; **Figure 6B**) which has the highest affinity to cortisol and a relatively low affinity for dexamethasone as determined through binding energy calculations (**Table 1**). The residues S371 and S373 are also part of an experimentally identified cortisol binding peptide (**Figure 3**), further increasing the likelihood of a disruption of cortisol-S1 binding due to their mutations in Omicron.

A67, H69, V70, T95, G142, V143, Y144, Y145, N211, L212 are present in the NTD of S1 protein. H69, V70 and Y144 deletions are known to disturb the interaction network observed in the wild type. This has direct implications in increased infectivity (as seen in Omicron: H69-, V70-, Y144-; Delta: E156-, F157-). V70, although near STP_134 (**Figure 6B**), is a part of a loop and may not affect the binding of HCY/DEX to this pocket. T95I has been reported to have no impact of S1 structure (43). Residue Y145 is close to STP_10, however the side chain faces away from the pocket (**Figure 6B**). A point mutation, Y145D may not affect the pocket and hence the interaction with the glucocorticoids. G339 is a residue that lies on a 3-turn helix in the proximity of STP_43. Residues in its proximity (P337, F338 and V341) are involved in HCY/DEX binding. G339D mutation can cause a change in the local secondary structure, which could affect the binding with the glucocorticoids. G446 was not present near any of the high affinity pocket. It is unlikely that G446S mutation alone could cause a distortion of the structure. Q493 and G496 are present near the groove of STP_87, STP_88 and STP_135 (**Table 6**), and the mutants Q493R and G496S will not perturb the above pockets which demonstrate high affinity to both the ligands (**Tables 1**, **3**, **4**). Q498 is not as close to any pockets as Q493 and G496, and its mutation to arginine, is unlikely to perturb any pockets, making all these mutants of S1 good targets of combinatorial therapy. Similarly, the side chain of N501 faces away from the high affinity pocket STP_152. Hence N501Y mutation might not perturb the pocket and hence could still bind to the glucocorticoids. H655Y is a variant in the linker region adjacent to S1/S2 furin cleavage site and does not affect the pockets identified in the study and can be a target for therapy.

## Discussion

**Figure 7:**
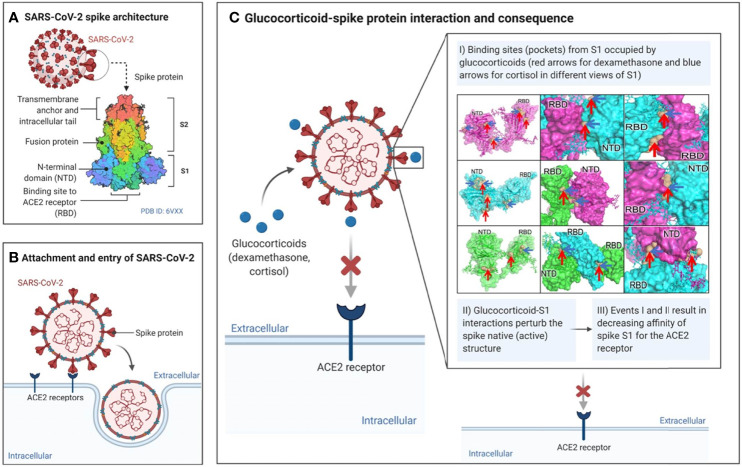
Summary of proposed mode of action of glucocorticoids in directly affecting SARS-CoV-2 – ACE2 interactions.

In this paper, we have established that the glucocorticoids cortisol and dexamethasone can directly bind to the SARS-CoV-2 surface glycoprotein S1 and inhibit its interaction with ACE2, a major receptor used by SARS-CoV-2 (and other coronaviruses) for infection of the host (**Figure 7**). Using molecular dynamics simulations and binding energy calculations, we identified all possible binding pockets for cortisol and dexamethasone on S1 – some of which we were able to further validate through the application of a limited proteolysis and mass spectrometry approach. Through interaction assays, we showed that cortisol and dexamethasone, separately and cooperatively inhibit the interaction between S1 and ACE2, through direct binding to wild type S1. Moreover, we examined the significance of our findings with regards to the ability of SARS-CoV-2 to mutate giving rise to variants of concern with enhanced virulence: Our data indicate that cortisol can disrupt the binding of a mutant S1 Beta variant (E484K, K417N, N501Y) to ACE2. Our *in silico* analyses indicate that the specific mutations in the highly infectious Delta and Omicron variants of concern may impact the binding of glucocorticoids to S1 and hence may also affect glucocorticoids inhibition of S1-ACE2 interactions. Interestingly, in the presence of cortisol, we found increased inhibition of the interaction between S1 and ACE2 by an anti-SARS-CoV-2 S1 human chimeric monoclonal antibody against the receptor binding domain. From a translational point of view, these data suggest therapeutic interventions involving combinations of glucocorticoids and S1 neutralizing antibodies. From a conceptual point of view, binding and disruption of the structural integrity of S1 could be a novel innate defence mechanism by which endogenous glucocorticoids may have contributed to asymptomatic SARS-CoV-2 infection –a topic that deserves further investigation.

Our *in silico* structural studies suggest that cortisol and dexamethasone bind SARS-CoV-2 S1 at multiple binding sites to synergistically disrupt S1 interaction with ACE2. Our thorough computational analysis of *in silico* acquired structural data, in the absence of experimental ligand-bound structures, present plausible high affinity ligand binding sites. The sites have high affinity to one ligand over another. Taken together, this in-depth analysis confirms the idea that the two ligands, cortisol and dexamethasone, can interact with different sites on spike S1 at the same time. Binding of these ligands to multiple sites at the same time could lock the spike monomers in an unfavorable conformation. For instance, the glucocorticoids binding to the pockets identified in the RBD-RBD interfaces could lead to a distorted RBD not being able to interact with the ACE2. This locked confirmation i.e., lack of plasticity and dynamics of the spike S1, would hinder its functionality and hence the propensity to invade the host.

Using limited proteolysis and mass spectrometry (a method of identifying ligand-specific binding regions in a protein), we confirmed a number of cortisol binding pockets that we had identified in our molecular dynamics simulation studies (e.g., HCY_8, HCY_29, HCY35, HCY 59, HCY_88, HCY_112, HCY_153, HCY_161). Cortisol binds to residues that form these binding pockets or their neighbouring residues that can either protect the binding site from proteolytic cleavage (which results in the peptide being detected only in the presence of cortisol) or facilitate proteolytic cleavage (which results in the peptide being detected only in the absence of cortisol), depending on the perturbation/conformational change cortisol induces at a binding site. These results confirm the direct binding of cortisol to SARS-CoV-2 S1 in addition to pin-pointing multiple binding sites as previously identified by our *in silico* analyses.

Noteworthy, the limited proteolysis and mass spectrometry enabled the positive identification of amino acids sequences of several sites on S1 which we predicted to be cortisol-binding sites. In many cases, site-directed mutagenesis would have been a valid alternative approach. However, site-directed mutagenesis would have been an unfeasible experiment in addressing glucocorticoids interactions with ACE2. Indeed, we found in excess of 52 different pockets in S1 with the minimum dimensions to position cortisol or dexamethasone and multiple residues of S1 that interact with these ligands. If we had pursued site directed mutagenesis, the number of combinatorial mutants of S1 to be produced, purified and folded would have simply been staggering. An expected result would have been mostly insoluble, unfolded S1 protein. Another anticipated result would have been the production of many biologically insignificant S1 mutants to which glucocorticoids might bind with increased or decreased affinities. However, there would have been no way of predicting if any of these mutants would fold in a conformation that binds to ACE2, which is a required control experiment. Further, many of these S1 mutants might not fold in a way that is compatible with formation of an infectious virus particle, thus they would be biologically completely irrelevant mutants. Therefore, and by contrast, we chose to analyze the Beta, Delta and Omicron S1 mutants which do produce viable and infectious viral variants, thus also biologically highly relevant (discussed further below).

Our biochemical studies demonstrate that cortisol and dexamethasone can directly inhibit the interaction between SARS-CoV-2 S1 and ACE2 at nanomolar concentrations, although concentrations higher than 1000 nM of either cortisol or dexamethasone demonstrated reduced efficacy. Our biochemical data suggests that cortisol and dexamethasone can cooperatively inhibit the interaction of SARS-CoV-2 S1 with ACE2 *via* their direct binding to SARS-CoV-2 S1 (not ACE2). Moreover, in the presence of cortisol, there is increased inhibition of the interaction between S1 and ACE2 by an anti-SARS-CoV-2 S1 human chimeric monoclonal antibody against the receptor binding domain. The effective concentration range for the cooperative actions of cortisol and dexamethasone or cortisol and anti- SARS-CoV-2 S1 antibodies was narrow displaying a characteristic ‘V’-shape of the %S1/ACE2 interaction vs. glucocorticoids concentration curve. Whether at high concentrations, cortisol and dexamethasone aggregate or bind low affinity sites on SARS-CoV-2 S1 which reduce their effects is unclear. However, from a therapeutic point of view, the characteristic ‘V’-shape of the %S1/ACE2 interaction vs. glucocorticoids concentration curve may not be a major limitation due to the dynamic nature of cortisol production, whose levels are well known to go up and down during the circadian cycle and in response to environmental stressors (47). Cortisol concentrations in the blood can range from 80 nM to 700 nM (48) which is within the range of concentrations which inhibit the interaction between SARS-CoV-2 S1 and ACE2 in our assays. Conceivably, in SARS-CoV-2 infected individual, there must be periods of time when the levels of endogenous cortisol reach those that allow for synergism and inhibition of binding of SARS-CoV-2 S1 to ACE2 which may result in some protection against viral infectivity via the ACE2 pathway. A similar positive effect on reduction of viral infectivity could be expected from synergism between endogenous SARS-CoV-2 antibodies and glucocorticoids.

Since its detection in Wuhan in December 2019, SARS-CoV-2 has mutated giving rise to variants of concern (such as the Alpha, Beta, Gamma, Delta and Omicron) that have numerous mutations in the RBD of spike protein SARS-CoV-2 S1 (41, 49). These mutant SARS-CoV-2 S1 variants display varying affinities towards ACE2 (49). Our results demonstrated that the interaction with ACE2 of the Beta variant SARS-CoV-2 S1 (with mutations K417N, E484K and N501Y) is inhibited by cortisol at the same concentrations with similar efficacies, compared to the wild type SARS-CoV-2 S1. This result was expected as the residues K417, E484 and N501 (mutated in Beta variant) were not found to be interacting with cortisol in our *in silico* analyses and also not found in experimentally identified cortisol-binding peptides. The increased number of mutations in the highly virulent Delta and Omicron SARS-CoV-2 S1 variants, however, include multiple mutated residues that are part of our identified cortisol/dexamethasone binding pockets. For instance, residues S371 and S373 in the RBD domain of chain A that are mutated in Omicron are present in the binding pocket STP_29 (high affinity to cortisol) as well as in an experimentally confirmed cortisol binding peptide. The mutation of these and other residues that were identified to be involved in binding cortisol/dexamethasone in Delta and Omicron SARS-CoV-2 S1 could arguably change the affinity of cortisol and dexamethasone for SARS-CoV-2 S1 and may consequently affect the inhibition of ACE2- SARS-CoV-2 S1 interaction by these glucocorticoids.

Our findings are potentially important as there are conflicting reports about the effectiveness of the use of glucocorticoids in treating COVID-19 patients. For instance, the UK-based Randomized Evaluation of COVID-19 Therapy trial found that dexamethasone 6 mg/d for 10 days results in reduction in 28-day mortality in patients receiving invasive mechanical ventilation at the time of randomization 17. However, a meta-analysis of 21,350 patients with COVID-19 concluded that the overall mortality was greater among COVID-19 patients who were receiving glucocorticoids than among patients that did not (50). The relationship between the mortality benefit of glucocorticoids and baseline levels of oxygenation, age, sex, comorbidities, and/or duration of symptoms and viral load are as unclear as the optimal timing for intervention (2). The mechanism underlying glucocorticoids reduction of mortality in some COVID-19 patients is unclear but may involve binding of glucocorticoids to their cell membrane receptor and intracellular signaling leading to genomic effects that may reduce inflammation (2). We investigated a new, complementary non-genomic mechanism involving direct binding of glucocorticoids to multiple sites on SARS-CoV-2 S1. Our observations suggest a possible role of endogenous glucocorticoids as a possible innate immunity mechanism related to direct interactions with SARS-CoV-2 S1 and unrelated to glucocorticoids anti-inflammatory actions. From our data, it is conceivable that exogenous glucocorticoids such as dexamethasone could improve innate immunity, at least, against certain variants of SARS-CoV-2 if given prophylactically to cohorts who are at high risk of severe COVID-19.

Infectivity studies are warranted to establish the role of endogenous glucocorticoids as a mechanism that may protect against SARS-CoV-2 infectivity. Although we were unable to conduct such studies, this merit further investigations. However, we have demonstrated that dexamethasone can downregulate the expression of ACE2 and TMPRSS2 on CD71+ erythroid cells which subsequently results in reduced permissibility of these cells to SARS-CoV-2 infection (51). Further experimentation beyond the capability of our labs is required to formulate cocktails of glucocorticoids and establish their influence on enhancing protection by anti-S1 antibodies. Similarly, studies are warranted to assess how the expression of ACE2 relative to other receptors affects infectivity in various relevant cell line models and if glucocorticoids exert an influence on the interactions of S1 with other CoV-2 receptors. Similarly, future research should address the potential interactions between glucocorticoids and the ever-increasing number of SARS-CoV-2 variants of concern, with numerous S1 mutations.

In conclusion, our biochemical and molecular dynamics simulation analyses unambiguously show that glucocorticoids bind to multiple pockets on SARS-CoV-2 S1 and induce significant conformation changes in S1 protein that can inhibit SARS-CoV-2 S1 binding to the cellular receptor ACE2. Conceivably, it should be possible to disrupt SARS-CoV-2 infection of the host by targeting multiple sites on SARS-CoV-2 S1 with glucocorticoid-like drugs. Similarly, a broadly applicable approach to disrupt pathogen/host interactions could be to target the pathogen at multiple sites. This notion is interesting as it contrasts with the conventional approach of inhibiting pathogen/host interactions with highly specific means such as neutralizing antibodies elicited by vaccines. Remarkably, we found that glucocorticoids can also cooperatively enhance the efficacy of a chimeric SARS-CoV-2 S1 neutralizing antibody against the RBD. Therefore, glucocorticoids- and neutralizing antibody-based inhibition of SARS-CoV-2/host interactions are compatible. We suggest that glucocorticoids may play a novel supportive role in protection against infectivity of coronaviruses (such as SARS-CoV-2) which use S1 for infection. Further research is warranted to establish whether direct interaction between endogenous glucocorticoids and SARS-CoV-2 S1 is an innate defence mechanism that may have contributed to mild or asymptomatic SARS-CoV-2 infection

## Data Availability Statement

The datasets presented in this study can be found in online repositories. The names of the repository/repositories and accession number(s) can be found below: http://www.ebi.ac.uk/pride, accession ID: PXD032937.

## Author Contributions

HS, RP, and CF-P conceived the study, designed the experiments and wrote the manuscript. HS and RP conducted the experiments and collected the data. HS and RP had equal contributions. All authors participated in the data analysis and performed critical revisions of the manuscript and approved the final version of the manuscript.

## Funding

Natural Sciences and Engineering Council of Canada (RES0034250). University of Alberta Hospital Foundation for Medical Research (RES0048483).

## Conflict of Interest

The authors declare that the research was conducted in the absence of any commercial or financial relationships that could be construed as a potential conflict of interest.

## Publisher’s Note

All claims expressed in this article are solely those of the authors and do not necessarily represent those of their affiliated organizations, or those of the publisher, the editors and the reviewers. Any product that may be evaluated in this article, or claim that may be made by its manufacturer, is not guaranteed or endorsed by the publisher.
