# Supplementary material for: Glucocorticoids Bind to SARS-CoV-2 S1 at Multiple Sites Causing Cooperative Inhibition of SARS-CoV-2 S1 Interaction With ACE2
Source: Front Immunol. 2022 Jun 15;13:906687. doi: 10.3389/fimmu.2022.906687 (PMC9242398; doi:10.3389/fimmu.2022.906687)
Supplement: Supplementary file 1 [file DataSheet_1.pdf]

Glucocorticoids bind to SARS-CoV-2 S1 at multiple sites causing cooperative inhibition of SARS-CoV-2 S1 interaction with ACE2

Hassan Sarker[1], Rashmi Panigrahi[1], Eugenio Hardy[2], Mark Glover[1], Elahi Shokrollah[3,4,5], Carlos Fernandez-Patron[1]*

[1]Department of Biochemistry, Faculty of Medicine and Dentistry, University of Alberta.

[2]Center of Molecular Immunology, P.O. Box 16040, Havana, Cuba.

[3]Department of Dentistry, Faculty of Medicine and Dentistry, [4]Department of Medical Microbiology and Immunology, [5]Department of Oncology, Faculty of Medicine and Dentistry, University of Alberta, Edmonton, AB T6G 2H7, Canada.

***Correspondence:**

Carlos Fernandez-Patron: cf2@ualberta.ca

Department of Biochemistry

Faculty of Medicine and Dentistry

University of Alberta

Edmonton, AB T6G 2H7, Canada

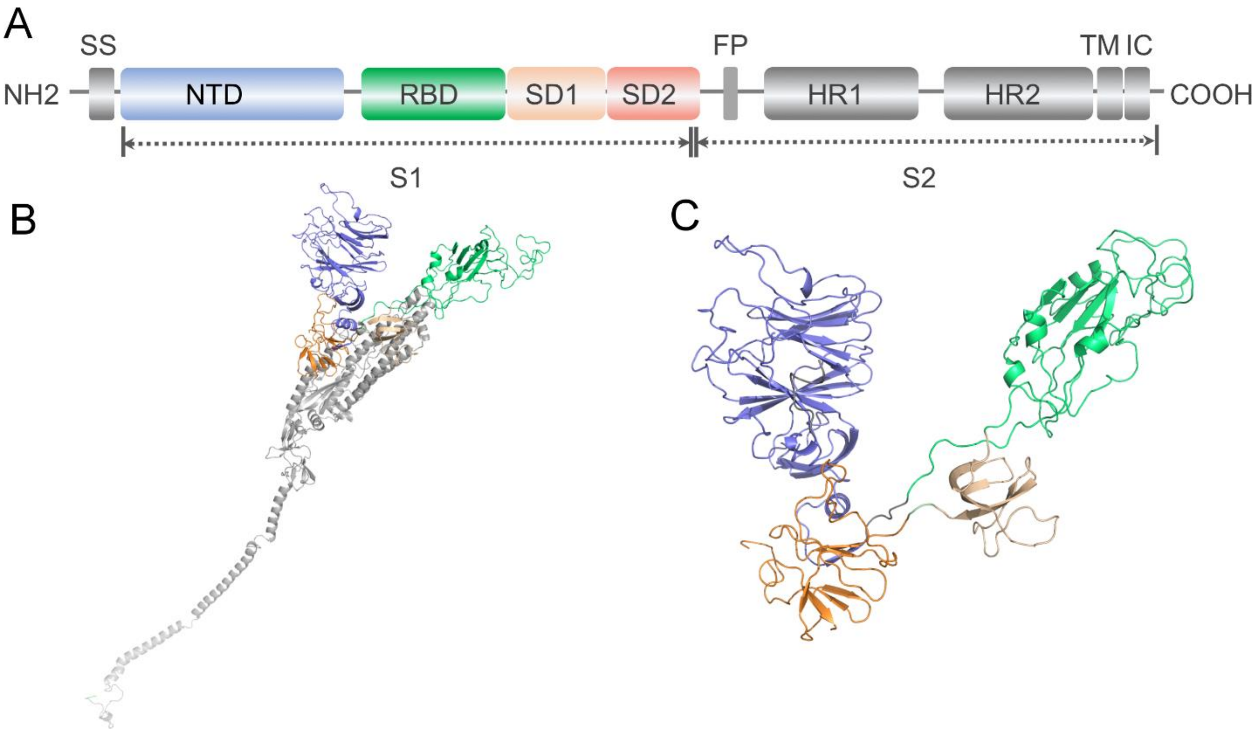

Supplementary figure 1: Overall structure of the SARS-CoV-2 spike (S) protein monomer.

A. Domain topology of the S monomer. NTD, N-terminal domain; RBD, receptor binding domain; SD1, subdomain 1; SD2, subdomain 2; FP, fusion peptide; HR1, heptad repeat 1; HR2, heptad repeat 2; TM, transmembrane region; IC, intracellular domain. **B**. 3D structural of S protein monomer obtained by modeling. Coordinates obtained from the Amaro lab database, University of California, San Diego (https://amarolab.ucsd.edu/covid19.php) **C**. Structure of S1 subunit of the spike protein demonstrating the 3D organization of NTD (slate) and RBD (green).

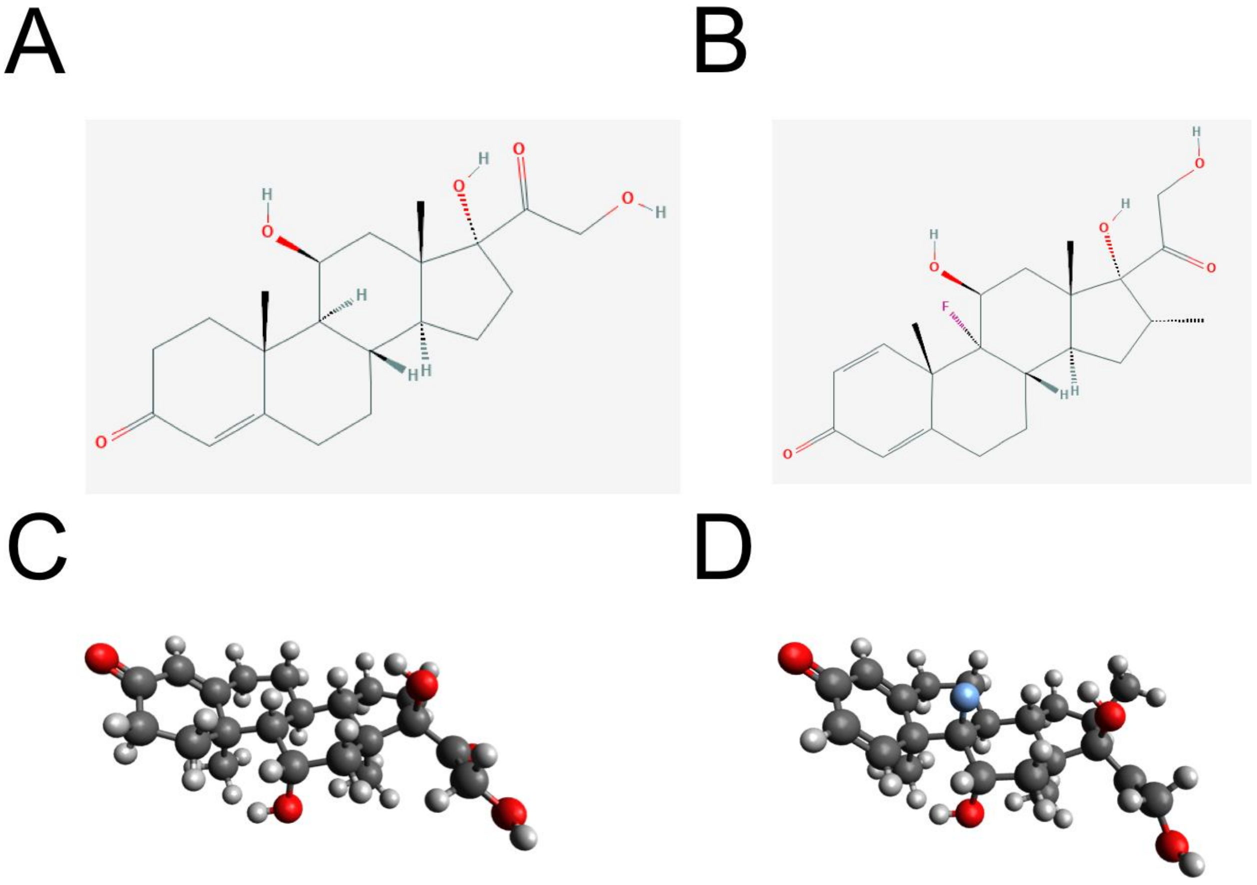

Supplementary figure 2: Structures of cortisol and dexamethasone. **A**. 2D structure of cortisol. **B**. 2D structure of dexamethasone. **C**. 3D structure of cortisol in ball and stick model. **D**. 3D structure of dexamethasone in ball and stick model.

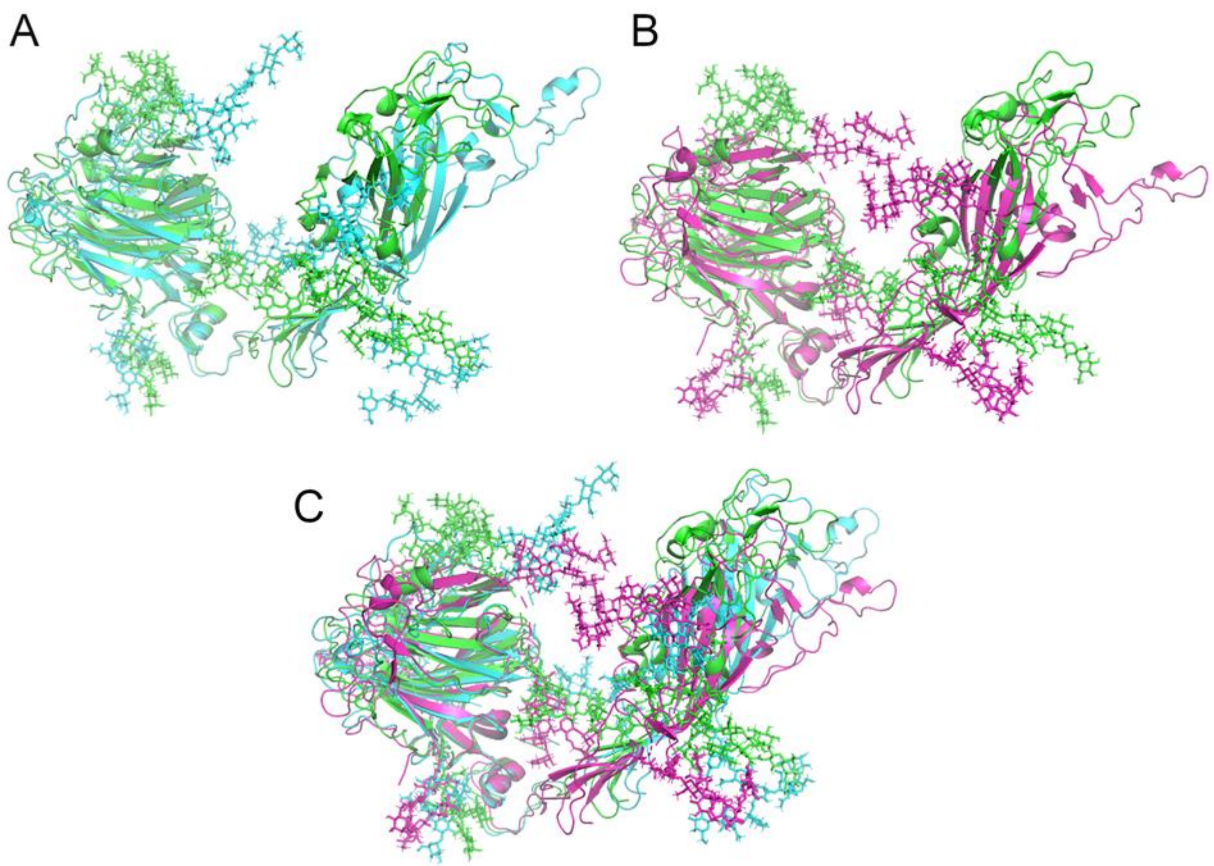

Supplementary figure 3: Analysis of dynamics between each monomer of SARS-CoV2 spike glycoprotein trimer. The final pdb from the 10 µs simulation was used for this analysis. The three monomeric structural components (chains- A, B and C) of the trimer were overlayed as pairs to visualize the dynamics in one with respect to the other. The protein chain is shown in cartoon and the glycan chains are shown in stick representations. (**A**) Overlay of chain A (green) and chain B (cyan); (**B**) Overlay of chain A (green) and chain C (magenta); (**C**) Overlay of the three chains. These overlays reveal that the glycan chains and the RBD domains are dynamic, while the NTD domains are relatively stable.

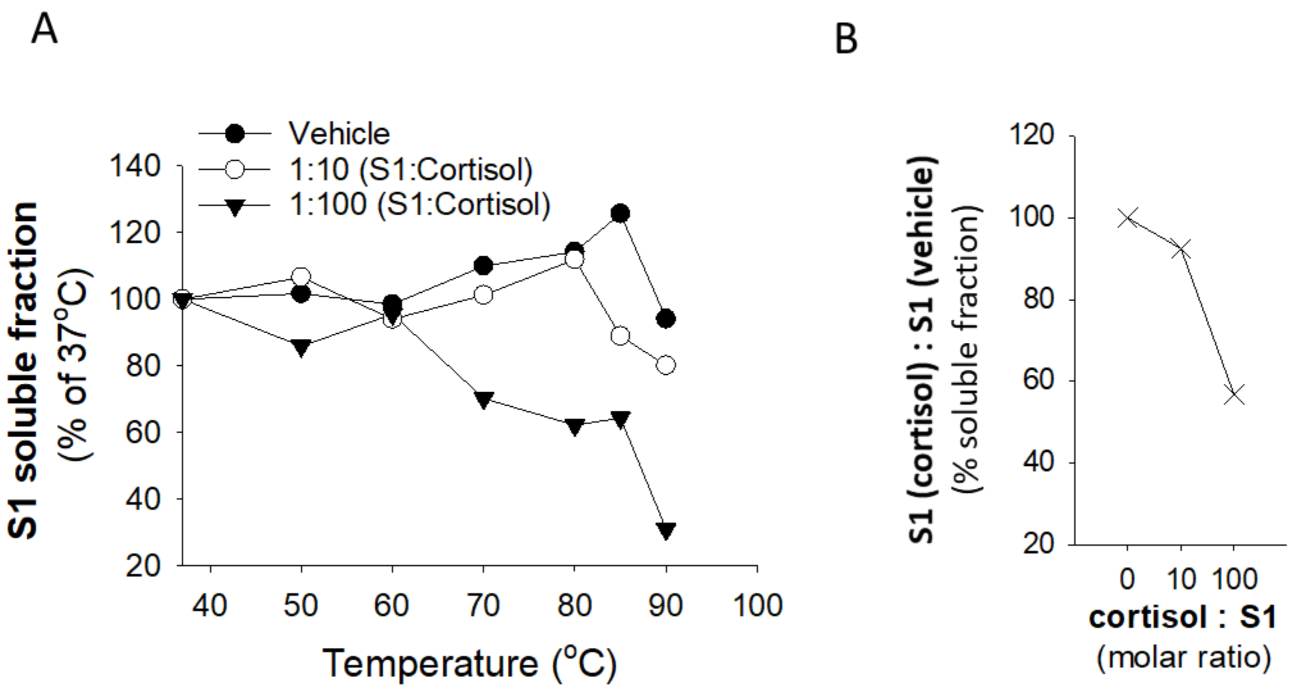

Supplementary figure 4: The effect of cortisol and dexamethasone on the thermal stability of SARS-CoV-2 S1. **(A)** Line plot showing solubility of SARS-CoV-2 S1 at increasing temperatures from 37°C to 90°C in the presence or absence of cortisol (10 nM or 100 nM). Soluble fraction was quantitated by densitometric analysis of SARS-CoV-2 S1 protein bands on an SDS-PAGE gel stained with Zn-Imidazole and plotted as a percentage of band intensity at 37°C (100% solubility). **(B)** Line plot showing solubility of SARS-CoV-2 S1 at 85C relative to 37C in the presence or absence of cortisol in cortisol:S1 molar ratios of 0:1, 10:1, 100:1.

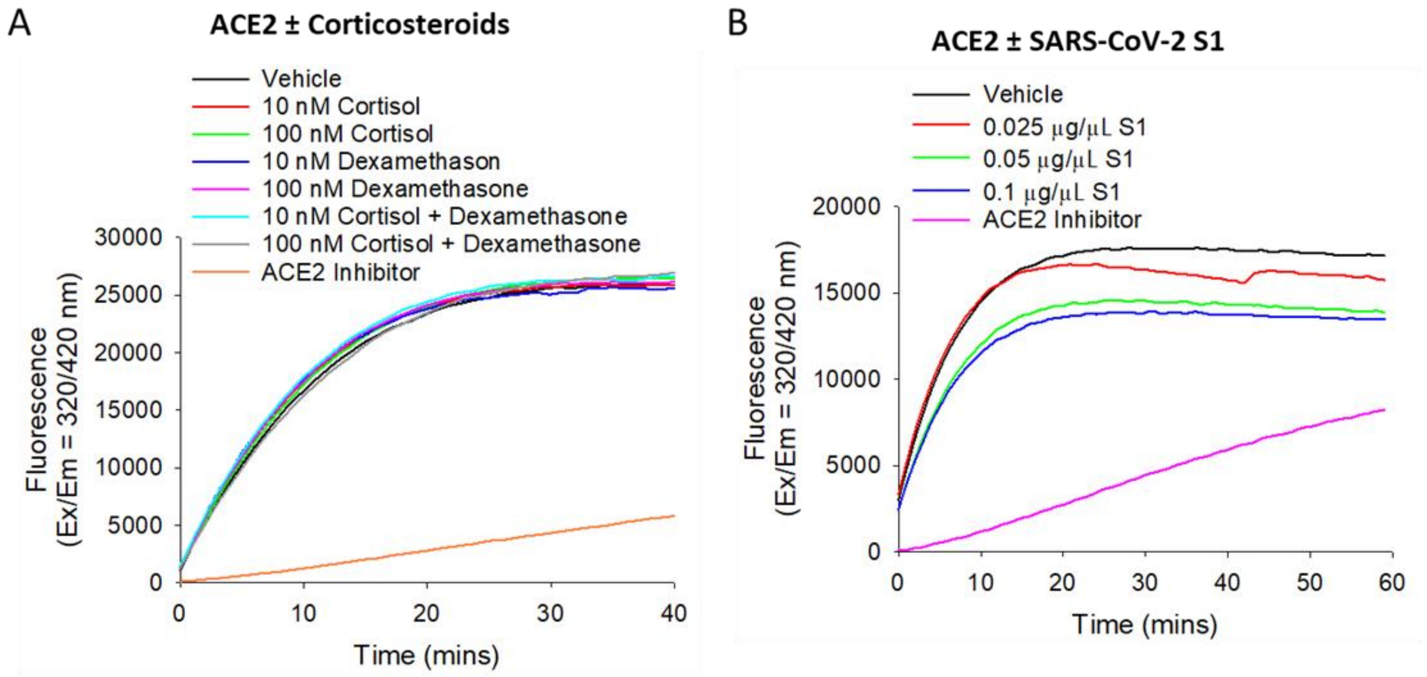

Supplementary figure 5: The effect of cortisol and dexamethasone on ACE2 activity. **(A)** Plots showing ACE2 activity (represented by rate of increase in fluorescence) in the presence or absence of increasing concentrations of cortisol or dexamethasone. Data shown are representative of triplicate independent experiments. **(B)** Plots showing ACE2 activity (represented by increase in fluorescence) in the absence or presence of increasing concentrations of SARS-CoV-2 S1. Data shown are representative of duplicate independent experiments.

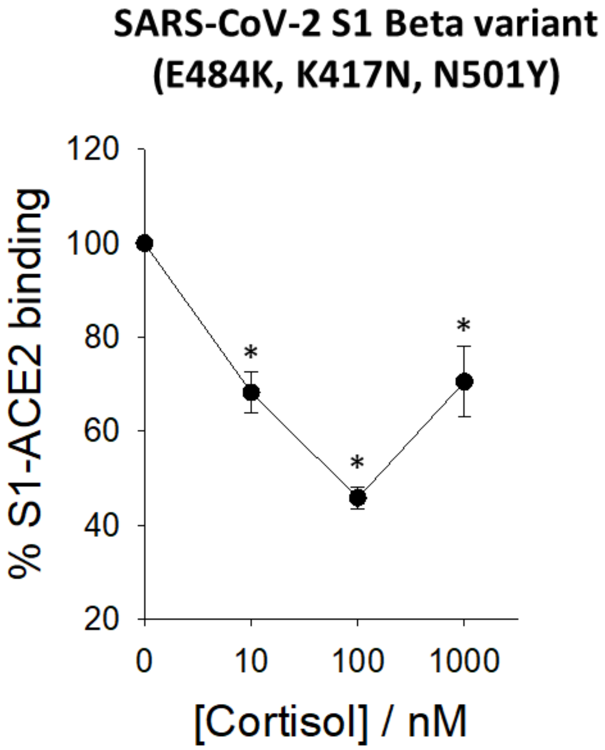

Supplementary figure 6: The effect of the cortisol on the binding of mutant SARS-CoV-2 S1 (E484K, K417N and N501Y, i.e., Beta variant) to ACE2. Plot showing % SARS-CoV-2 S1-ACE2 binding in the presence of increasing concentrations of cortisol. Each data plot was normalized to vehicle (100% S1-ACE2 binding). Each data plot was normalized to vehicle (100% S1-ACE2 binding). Data were measured in 2 independent replicates and are presented as mean ± standard error. *****P<0.05 vs vehicle (0 nM) (1-way ANOVA).

Supplementary movie: SARS CoV2 Spike protein trimer with predicted high affinity pockets using F pocket. The pocket (in white) is the unique to each S1 monomer and the pockets (in blue) are formed between domains of different S1 monomers.

Supplementary Table 1: Full list of peptides identified by mass spectrometry (MS) following limited proteolysis of SARS-CoV-2 S1 incubated with vehicle or cortisol. Peptides highlighted blue are unique peptides only present in either vehicle or cortisol sample due to cortisol binding at these sites either facilitating or preventing proteolysis of these sites. These experimentally identified binding sites of cortisol coincide with those identified by our in-silico molecular simulation validating our hypothesis that cortisol (and possibly other glucocorticoids) can bind to multiple pockets on S1 which is likely to cause S1 denaturation and decreased affinity for its receptor, ACE2. Y: peptide detected by MS in the sample; N: peptide not detected by MS in the sample. Supplementary proteomics raw data are provided as a supplementary excel spread sheet.

Peptides	Vehicle	Cortisol
TFLLK	Y	Y
EFVFK	Y	N
DLICAQK	N	Y
NIDGYFK	Y	Y
IADYNYK	Y	Y
CYGVSPTK	Y	Y
HTPINLVR	Y	Y
SNLKPFER	Y	Y
VQPTESIVR	Y	Y
NLREFVFK	Y	Y
SWMESEFR	Y	Y
DIADTTDAVR	Y	Y
GIYQTSNFR	Y	Y
FQTLLALHR	Y	Y
FASVYAWNR	Y	Y
KSNLKPFER	Y	Y
FLPFQQFGR	Y	Y
VGGNYNYLYR	Y	Y
GWIFGTTLDSK	Y	Y
SFIEDLLFNK	Y	N
FASVYAWNRK	Y	Y
GVYYPDKVFR	Y	Y
LIANQFNSAIGK	Y	N
NIDGYFKIYSK	Y	N
VYSTGSNVFQTR	Y	Y
TQLPPAYTNSFTR	Y	Y
NLNESLIDLQELGK	N	Y
SFTVEKGIYQTSNFR	Y	N
VVVLSFELLHAPATVCGPK	Y	Y
FDNPVLPFNDGVYFASTEK	Y	Y
VVVLSFELLHAPATVCGPKK	Y	Y
CVNFNFNGLTGTGVLTESNK	N	Y

LPDDFTGCVIAWNSNNLDSK	Y	Y
CVNFNFNGLTGTGVLTESNKK	Y	Y
RFDNPVLPFNDGVYFASTEK	Y	Y
VCEFQFCNDPFLGVYYHK	Y	Y
ISNCVADYSVLYNSASFSTFK	Y	Y
RISNCVADYSVLYNSASFSTFK	N	Y
LNDLCFTNVYADSFVIRGDEVR	N	Y
